# Mitotypes Based on Structural Variation of Mitochondrial Genomes Imply Relationships With Morphological Phenotypes and Cytoplasmic Male Sterility in Peppers

**DOI:** 10.3389/fpls.2019.01343

**Published:** 2019-10-24

**Authors:** Yeong Deuk Jo, Hea-Young Lee, Na-Young Ro, Sang Hoon Kim, Jin-Baek Kim, Byoung-Cheorl Kang, Si-Yong Kang

**Affiliations:** ^1^Radiation Breeding Team, Advanced Radiation Technology Institute, Korea Atomic Energy Research Institute, Jeongeup, South Korea; ^2^Department of Plant Science and Vegetable Breeding Research Center, Seoul National University, Seoul, South Korea; ^3^National Agrobiodiversity Center, National Academy of Agricultural Science, Rural Development Administration, Jeonju, South Korea

**Keywords:** mitotype, structural variation, pepper, cytoplasmic male sterility, domestication

## Abstract

Plant mitochondrial genomes characteristically contain extensive structural variation that can be used to define and classify cytoplasm types. We developed markers based on structural variation in the mitochondrial genomes of fertile and cytoplasmic male sterility (CMS) pepper lines and applied them to a panel of *Capsicum* accessions. We designed a total of 20 sequence characterized amplified region (SCAR) markers based on DNA rearrangement junctions or cytoplasm-specific segments that did not show high similarity to any nuclear mitochondrial DNA segments. We used those markers to classify the mitotypes of 96 *C. annuum* accessions into 15 groups. Precise genotyping of other *Capsicum* species (*C. frutescens*, *C. chinense*, and *C. baccatum*) was hampered because of various stoichiometric levels of marker amplicons. We developed a multiplex PCR system based on four of the markers that efficiently classified the *C. annuum* accessions into five mitotype groups. Close relationships between specific mitotypes and morphological phenotypes implied that diversification or domestication of *C. annuum* germplasm might have been accompanied by structural rearrangements of mitochondrial DNA or the selection of germplasms with specific mitotypes. Meanwhile, CMS lines shared the same amplification profile of markers with another mitotype. Further analysis using mitochondrial DNA (mtDNA) markers based on single-nucleotide polymorphisms (SNPs) or insertions and deletions (InDels) and CMS-specific open reading frames (*orf*s) provided new information about the origin of the CMS-specific mitotype and evaluation of candidates for CMS-associated genes, respectively.

## Introduction

In comparison to animals, the rate of nucleotide substitution in mitochondrial genome is much slower in most plant species except for several independent lineages that show highly accelerated rate, whereas evolution of genome structure is much faster and on a large scale (Kress et al., 2005; Sloan et al., 2012; Wolfe et al., 1987). Although mitochondrial genes, such as *cytochrome c oxidase 1* (*CO1*), have been widely used as bar code sequences to investigate biological diversity in a broad range of animal species, their application in plants has been hampered because of the slow rate of substitution (Kress et al., 2005). In contrast, changes in genome structure occur frequently and extensively in plants, even within species, and therefore can be used to characterize and classify mitochondrial haplotypes (mitotypes) at the intraspecific level (Davila et al., 2011; Kubo et al., 2011). Besides its importance in analyses of genetic diversity, mitotype classification is useful for selecting accessions carrying genes associated with cytoplasmic male sterility (CMS), which originates from DNA rearrangements (Kim and Kim, 2005; Kim et al., 2007a; Xie et al., 2014; Heng et al., 2017).

Structural variation in plant mitochondrial genomes results from DNA rearrangements and substoichiometric shift (SSS), or changes in stoichiometric levels of subgenomic molecules that constitute the mitochondrial genome. DNA rearrangements are caused by recombination *via* repeat sequences and nonhomologous end-joinings (NHEJs) that follow double-strand breaks (Davila et al., 2011; Tang et al., 2017). Diverse parental and recombinant molecules coexist in mitochondria, maintaining a certain relative ratio between them, but SSS can cause rapid amplification or loss of specific molecules (Janska et al., 1998; Arrieta-Montiel et al., 2001; Feng et al., 2009; Chen et al., 2011). Those extensive changes can be caused by wide crosses, *in vitro* culture, or the suppression or mutation of genes involved in the surveillance of mitochondrial DNA (mtDNA) recombination (Hanson and Bentolila, 2004; Sandhu et al., 2007; Shedge et al., 2007).

Molecular markers to detect structural variation in mtDNA have been developed mostly from DNA structures spanning CMS-associated genes. Such markers have been applied to select for lines containing a specific CMS cytoplasm or to elucidate the evolutionary processes that resulted in the emergence of the CMS-associated genes (Kim and Kim, 2005; Kim et al., 2007a; Tang et al., 2017). Recently, whole mitochondrial genome sequences have been analyzed in at least two different cytoplasms of several crop species, making it feasible to develop mitochondrial genome-wide DNA markers for the simultaneous classification of different mitotypes of those species (Allen et al., 2007; Liu et al., 2011; Tanaka et al., 2012). For example, Heng et al. (2017) developed 12 mitotype-specific markers based on structural variation among six sequenced mitochondrial genomes of *Brassica napus*. Those markers enabled the successful identification of mitotypes for six different cytoplasms.

Complete sequences of nuclear and mitochondrial genomes have been reported for pepper (Jo et al., 2014; Kim et al., 2014). Molecular markers dispersed evenly across the nuclear genome were selected and applied to a large panel of *Capsicum* accessions (3,821 accessions) for the construction of a core collection and phylogenic analysis (Lee et al., 2016). On the other hand, mtDNA markers have been developed mostly from a restricted region of the mitochondrial genome close to the CMS candidate genes, such as *orf507*, *Ψatp6-2*, and *orf300a* (Kim and Kim, 2005; Kim and Kim, 2006; Gulyas et al., 2010; Wang et al., 2019). Those markers usually classify mitochondrial genomes as one of two cytoplasm types because they were developed to select CMS cytoplasm. Therefore, the existing mtDNA markers are not adequate for studies of the structural diversity of the mitochondrial genome among germplasms.

A more extensive classification of pepper mitochondrial genomes would be useful not only to select CMS cytoplasm but also to investigate relationships between mitotypes and phenotypes. The *orf507* and *Ψatp6-2* genes were implicated as candidate CMS genes based on the induction of male sterility in heterologous transformation experiments using *orf456* (the 5’ part of *orf507*) and on a change in the transcription pattern of *Ψatp6-2* in the presence of the *Restorer-of-fertility* (*Rf*) gene, respectively (Kim and Kim, 2006; Kim et al., 2007b). It is possible, however, that other genes are involved in CMS induction because the induction of sterility by *orf507* and *Ψatp6-2* has not been confirmed by direct transformation experiments. Moreover, *orf507* is present in some pepper lines with normal cytoplasm in high copy number (Jo et al., 2009; Ji et al., 2014). Jo et al. (2014) and Wang et al. (2019) presented lists of CMS candidate genes based on comparative analysis of mitochondrial genomes between a CMS and a normal pepper accession, respectively. Especially, Wang et al. (2019) performed expression analysis on candidate genes and applied a molecular marker from one of the candidates (*orf300a*) to their breeding lines to show its corelationship with CMS. However, other candidate genes suggested newly in this analysis still could not be excluded because the marker test was not performed for them. In addition, diversity of test lines was not represented. The evaluation of CMS candidate genes using diverse pepper accessions with classified mitotypes may supplement these studies. Except for CMS, possible relationships between mitotypes and phenotypes at the intraspecific level have never been examined in pepper, although one study demonstrated a relationship between haplotypes of plastid DNA-derived markers and fruit shapes (Jo et al., 2011). A comparative analysis of mitotypes and phenotypes may help determine the lineages of germplasms with specific phenotypes and shed light on a possible role of mitochondria in specific phenotypes.

We developed molecular markers based on structural variations between two complete pepper mitochondrial genomes and applied them to a subset of core-collection *Capsicum* accessions. Our goal was to identify and classify *Capsicum* mitotypes and analyze their relationships with CMS and other phenotypes.

## Materials and Methods

### Plant Materials

We used two *Capsicum annuum* lines and their progenies to confirm the specific amplification of markers. The Korean landrace Jeju Jaerae contains normal cytoplasm and is the same line used in a previous study for mitochondrial genome sequencing (Jo et al., 2014). The breeding line Milyang K has CMS cytoplasm but is fertile because it also has the *Rf* gene. The Milyang K cytoplasm shares the same origin as the CMS line “FS4401,” which was also used previously for mitochondrial genome sequencing (Jo et al., 2014). We created Jeju Jaerae and Milyang K progenies from reciprocal crosses.

We used 12 *Capsicum* accessions for the preliminary application of markers: three *C. annuum* CMS breeding lines (Chungyang A, Bukang A, and KR2 A), three fertile and pungent *C. annuum* accessions [CM334 (the material used in whole genome sequencing by Kim et al., 2014)), Yuwol, and Chilsung], two fertile and nonpungent bell-type *C. annuum* accessions (Yolo Wonder and Del ray bell), and one accession each of *C. chinense* (Habanero), *C. frutescens* (AC08-026), *C. baccatum* (AC08-062), and *C. chacoense* (AC08-075). Among those, at least, CM334, Yuwol, Chilsung, and Yolo Wonder have been proven to contain normal cytoplasm. We used a total of 96, 16, 16, and 16 accessions of *C. annuum*, *C. chinense*, *C. frutescens*, and *C. baccatum*, respectively, for the application of markers to classify mitotypes. Those accessions were mostly a subset of accessions from a core collection that was constructed based on phenotypes and genotypes determined by nuclear single-nucleotide polymorphism (SNP) markers (Lee et al., 2016).

### Design and Application of Sequence Characterized Amplified Region Markers

**Figure 1:**
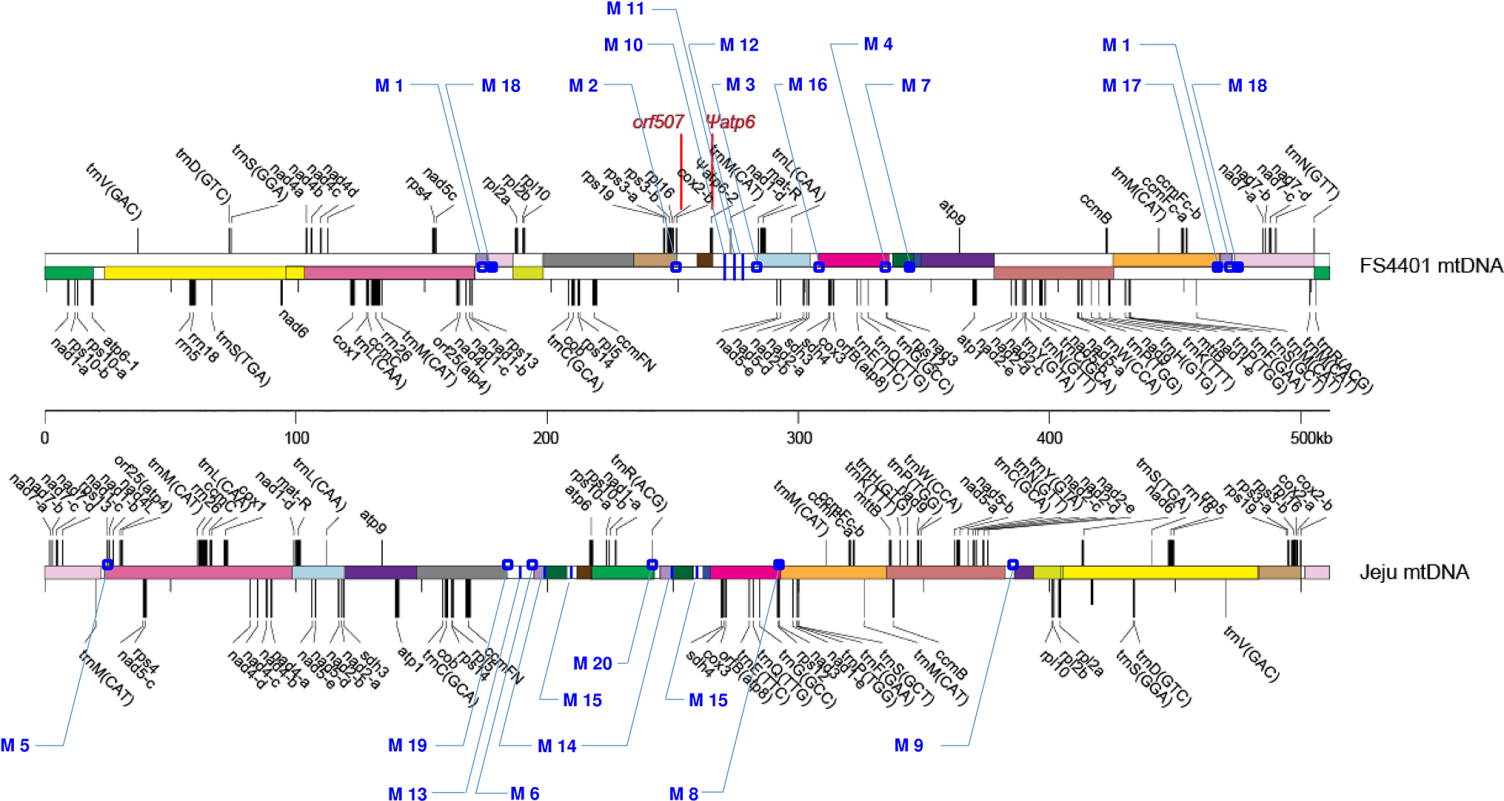
Locations of the 20 SCAR markers in the mitochondrial genomes of FS440 and Jeju Jaerae. The illustration of the genome structures of the two lines was adopted from Jo et al. (2014). Rectangles with the same color indicate syntenic DNA blocks between two lines; those with white colors show cytoplasm-specific segments. Open and closed dots represent locations of markers that were developed from DNA regions spanning junctions between syntenic blocks and from those located between a syntenic block and a cytoplasm-specific segment, respectively. Blue vertical lines refer to locations of markers that were developed from DNA regions inside cytoplasm-specific segments. The locations of *orf507* and *Ψatp6-2* are shown in red. Jeju Jaerae is abbreviated as “Jeju.”

Two types of DNA sequences were identified from the complete mtDNA sequences of Jeju Jaerae (normal cytoplasm) and FS4401 (CMS cytoplasm) (**Figure 1**). The first type was sequences (2 kb) that surround the junctions between two sequentially located syntenic blocks of mtDNA [DNA regions (>2 kb) that can be aligned between Jeju Jaerae and FS4401] or between one syntenic block and one sequentially located sequence that is unique to one of the two cytoplasms. Among them, we used only the sequences surrounding junctions between nonoverlapping syntenic blocks (in other words, junctions that are not the result of DNA rearrangements involving repeated sequences). The second type was sequences (>1 kb) located inside a larger sequence that is unique to one of the cytoplasms. In the next step, we performed BLAST against to CM334 genome (http://peppergenome.snu.ac.kr; E value < 10^-5^) and excluded any sequences with high similarity to multiple sequence in CM334 genome to rule out nuclear mitochondrial sequences (Numts). To amplify the selected sequences, primers were designed to have a T_m_ close to 59°C (**Supplementary Table S1**).

To confirm the specific amplification in one of the two lines, we applied them to four individuals of Jeju Jaerae and Milyang K, respectively. In addition, the markers were applied to two progenies from reciprocal crosses between Jeju Jaerae and Milyang K to ensure that the amplicons in one of parental lines were totally from mtDNA, not from Numts. Only the markers that specifically amplified one of the two cytoplasm types were selected for further study.

### DNA Extraction and PCR

DNA was extracted from young leaves using a cetyltrimethylammonium bromide (CTAB) protocol described previously (Park et al., 2009). The DNA concentration was determined with a NanoDrop 1000 spectrophotometer (NanoDrop Technologies, Wilmington, DE, USA). For each sequence characterized amplified region (SCAR) marker, PCR was carried out in a total volume of 20 μl containing 50 ng DNA, 200 μM deoxynucleotide (dNTP) solution mix, 2 μl 10× Taq DNA polymerase buffer, 5 μM each primer, and 0.75 units Taq DNA polymerase (Takara Bio, Inc., Kusatsu, Japan). The PCR conditions were as follows: 35 cycles of 98°C for 10 s, 59°C for 30 s, and 72°C for 100 s, followed by 72°C for 10 min. The sequences of the primers used to amplify *Ψatp6-2* were as follows: 5’-TGCATCTCGCTATTAACCAC-3’ and 5’- GTAGTTCATTCGGACCTAGTAG-3’. Except for annealing temperature during amplification (57°C), the other PCR conditions and reagents were the same as those used for the SCAR markers.

### Amplicon Detection and Genotype Scoring

Amplicons produced by each marker were loaded onto 0.8% agarose gel containing SafeView^TM^ Classic (Applied Biological Materials, Vancouver, Canada). After electrophoresis for 20 min at 100 V, DNA bands were visualized under UV-irradiation with the UVIdoc HD5 gel documentation system (Uvitec Ltd., Cambridge, UK). For each SCAR marker, we defined the *C. annuum* accessions that produced faint DNA bands as “low (L)” and those that showed saturation of amplicon yields as “high (H).” For the accessions of other *Capsicum* species, we defined those that show moderate or slight amplification as “uncertain (U)” and those with saturation of amplicon yields as “certain (C).” We designated the accessions that did not yield any detectable amplicons for a given marker as “none (N).” PCR amplification and genotyping were repeatedly performed two times with the same plant material for each accession. More replications were conducted to decide the final genotypes of accessions in cases that genotyping results were not consistent in two replications (**Supplementary Table S2, 3**). Because variation in mitochondrial genomes may even present among siblings (Elansary et al., 2010), three individuals for each accession were analyzed in application of four markers that were used for development of multiplex marker and classification of five mitotypes (see below; **Supplementary Table S4**) to check reproducibility.

### Multiplex PCR

Among four SCAR markers that were selected for multiplex PCR, two markers were redesigned to ensure enough length differences between amplicons for visual discrimination of bands on agarose gel (**Supplementary Table S4**). Multiplex PCR was carried out in a total volume of 20 μl containing 50 ng DNA, 200 μM dNTP solution mix, 2 μl 10× Taq DNA polymerase buffer, 2.5 μM each of the eight primers for the four selected SCAR markers, and 0.75 units Taq DNA polymerase (Takara Bio, Inc., Kusatsu, Japan). The PCR amplification conditions were 35 cycles of 98°C for 10 s, 59°C for 30 s, and 72°C for 100 s, followed by 72°C for 10 min.

### Calculation of Polymorphism Information Content

Polymorphism information content (PIC) values for each SCAR marker and the multiplex PCR marker (MPM1) were calculated according to following formula:

$$PIC_{i}=\sum_{j=1}^{n}P_{ij}{\text{​}}^{2}$$

where *PIC* *_i_* is the PIC of marker *i*, and *Pij* is the relative frequency of *j*th allele of marker *i*.

### Sequence Analysis of the SCAR Marker Amplicons

We performed Sanger sequencing of 35 amplicons from the *Capsicum* accessions to confirm specific amplification of the target sequences. Twenty-three of the selected amplicons were critical to the specification of minor mitotypes. We randomly selected 12 additional amplicons from accessions that were classified as different mitotypes. In the Sanger sequencing, amplicons were directly sequenced without subcloning. We removed the sequences at 5’ and 3’ ends of the amplicons that showed low sequencing quality and used the remaining internal sequences for our analysis.

### Classification by Nuclear Markers and Phenotype Analysis of *C. annuum* Accessions

We used data collected previously by Lee et al. (2016) to classify the *C. annuum* accessions by nuclear markers and phenotype. Lee et al. (2016) applied 48 SNP markers developed by transcriptome sequencing to a large *Capsicum* collection comprising 3,821 accessions. They divided the accessions into 10 clusters based on the SNP markers using the STRUCTURE program ver.2.3.4. They then created a core collection containing 240 accessions based on the 48 SNP markers and 32 phenotypic traits. The phenotype data were obtained in 2014 at a research farm (Seoul National University, Suwon, Republic of Korea) as described previously (Lee et al., 2016). The measurement of phenotype was performed in three independent individuals, and the average values were used for further analysis. We determined the differences between the mean values of groups classified by mtDNA or nuclear markers using Duncan’s multiple range tests (*P* < 0.05) following one-way ANOVA with the IBM SPSS statistics software (IBM Corp., Armonk, NY, USA).

### Development and Application of Markers Based on SNPs or InDels in mtDNA

The mtDNA sequence of Jeju Jaerae was split into 1-kb sequences and screened against the FS4401 mtDNA sequence using BLAST to identify sequences in the Jeju Jaerae mtDNA containing at least three SNPs or InDels. We then selected the sequences that did not show high similarity to CM334 nuclear DNA. Among the selected sequences, six were randomly selected and amplified in Milyang K, Jeju Jaerae, Yolo Wonder, and Habanero DNA. Finally, we selected the sequences that produced amplicons in all four pepper lines for marker development. From one of the sequences, we obtained a marker (mt-217) based on a portion of sequence obtained by direct Sanger sequencing from the reverse primer. We used 275-bp sequences that could be accurately analyzed by single Sanger sequencing reactions in all accessions for comparative analysis (**Supplementary Figure S1A**). In addition, we obtained one SCAR marker based on an InDel in another selected sequence (mt-227; **Supplementary Figure S1B**). We applied those two markers and a SCAR marker from plastid DNA (PepRpl; Jo et al., 2011) to the 144 *Capsicum* accessions. Information about the primer sequences and annealing temperatures is described in **Supplementary Table S5**.

### Analysis of Candidates for CMS-Associated Open Reading Frames

We screened the FS4401 mitochondrial genome for open reading frames (*orf*s) predicted to encode hypothetical proteins 50 amino acids or longer in length. We then used BLAST to identify which of those FS4401 *orf*s are truncated or absent in Jeju Jaerae. We used the DAS-TMfilter server (http://www.enzim.hu/DAS/DAS.html) to predict the presence of transmembrane domains in the hypothetical proteins encoded by each *orf*. We mapped transcriptome sequencing reads from a CMS *C. annuum* line (121A) and its near-isogenic restorer line (121C; Liu et al., 2013) obtained from the NCBI Gene Expression Omnibus to the selected *orf*s using CLC Workbench 8.5 (CLC Bio, Aarhus, Denmark), setting the threshold of similarity between the reads and the *orfs* to 90%. In the study of Liu et al. (2013), RNAs were isolated from mixture of floral buds in five developmental phases and annealed with biotinylated random primers after fragmentation for library preparation. We selected the *orf*s with RPKM (reads per kilobase of transcript per million mapped reads) values higher than three in 121A and amplified them in the 144 *Capsicum* accessions using the primers listed in **Supplementary Table S6**. To validate the difference in expression levels of candidate *orf*s in the presence of *Rf*, real-time quantitative reverse transcription PCR (qRT-PCR) was performed. A hybrid cultivar, Chungyang (S, *Rfrf*), and a CMS line, Bukang A (S, *rfrf*), were used as plant materials. Total RNA was extracted using Qiagen RNeasy mini kit (Qiagen, Dusseldorf, Germany) according to the manufacturer’s instruction from anthers of floral buds that were 4 mm in size and pooled from each plant. Additional DNase treatment was performed to completely remove mitochondrial DNA using RNase-free recombinant DNase I (Takara Bio, Otsu, Japan). First-strand cDNA was synthesized from random primers (random nonamers) using ReverTra Ace -α-^®^ reverse transcriptase kit (Toyobo, Osaka, Japan). The qRT-PCR was performed with mixture prepared using SYBR^®^ Premix Ex Taq II kit (Takara Bio, Otsu, Japan) by CFX96 Touch Real-Time PCR Detection System (Bio-Rad, Hercules, CA, USA). The primer sequences used for five candidate genes were shown in **Supplementary Table S6**. The amplification condition was as follows: 50°C for 10 min, 95°C for 10 min, followed by 60 cycles of 95°C for 15 s, 57.5°C for 15 s, and 72°C for 30 s. Standard curve of qRT-PCR was obtained for each primer combination using serially diluted (1, 1:5, 1:25, and 1:125) cDNA (pool of cDNA from all individuals used for qRT-PCR) by CFX Manager^TM^ Software (Bio-Rad, Hercules, CA, USA) and was used to determine qRT-PCR efficiency and relative expression level. The expression level of each candidate was normalized against a mitochondrial rRNA gene, *rrn26*. The sequences of primers used for *rrn26* were 5′-CCAGTGGAAGGTTTTCTCGTTC-3′ and 5′-CTCGGCCCGATCATCCAATTC-3′. The expression level of another rRNA gene, *rrn18*, was analyzed additionally for comparison (primer sequences: 5′-GGGTTGAAAGTGAAAGCCGC-3′and 5′-CATGGACTACCAGGGTATCTAATCC-3′). The qRT-PCR analysis was performed with three biological replicates. The statistical difference between groups was determinedby a *t*-test.

## Results

### Development of Molecular Markers for the Detection of Structural Variation in mtDNA

**Figure 2:**
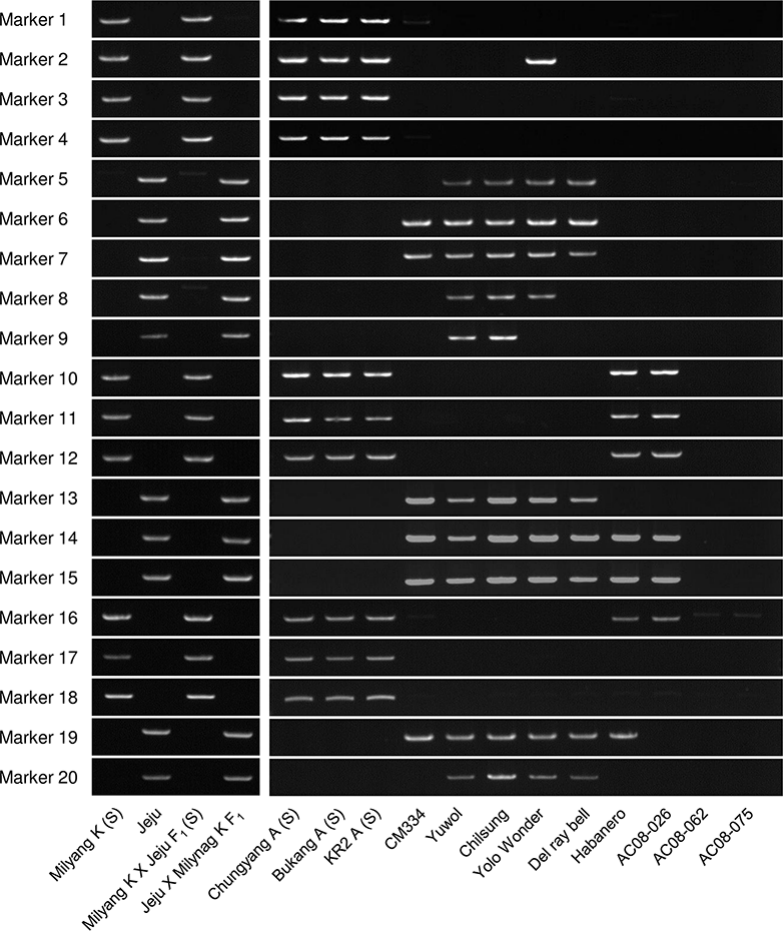
Application of the 20 SCAR markers to a panel of *Capsicum* accessions, including CMS lines. Jeju Jaerae is abbreviated as “Jeju.”

The Jeju Jaerae (normal cytoplasm) and FS4401 (CMS cytoplasm) mitochondrial genomes consist of both syntenic blocks of DNA and regions of DNA that are unique to one of the two cytoplasms (Jo et al., 2014; **Figure 1**). We collected sequences that surround DNA rearrangement junctions or are unique to one of the two cytoplasms and used BLAST to compare them against the CM334 nuclear genome to select sequences that have no homologous Numts. We used the selected sequences to design 42 primer combinations, including 31 from rearrangement junction-containing sequences (13 and 18 from Jeju Jaerae and FS4401, respectively) and 11 from cytoplasm-unique sequences (five and six from Jeju Jaerae and FS4401, respectively). We used those primers to amplify DNA from Jeju Jaerae and Milyang K (known to share the same origin of CMS cytoplasm with FS4401). A total of 21 primer pairs resulted in specific amplification in one of these lines. In application to two progenies from reciprocal crosses between Jeju Jaerae and Milyang K, one primer pair showed amplification in both progenies, indicating that a portion of parental line-specific amplicon was from Numt. Other 20 primer pairs, 10 for each cytoplasm, showed clear and specific amplification only in one of the two cytoplasms (**Supplementary Table S1; Supplementary Figure S2**; **Figure 2**). Thus, the amplicons resulting from those 20 primer pairs do not have homologous Numts in Jeju Jaerae and Milyang K and are either absent or present in very low copy number in the cytoplasm in which they did not produce detectable amplicons. We used those amplicons as SCAR markers in further applications to detect structural variations in mtDNA.

### Application of the SCAR Markers to *Capsicum* Accessions

We screened 12 *Capsicum* accessions (**Figure 2**) for the 20 SCAR markers. In *C. annuum*, the markers that were amplified in FS4401 were specifically amplified in the other CMS lines, except for marker 2, which was amplified in the normal cytoplasm line Yolo Wonder (Wang et al., 2004; **Figure 2**). In contrast, the markers that were amplified in Jeju Jaerae were not amplified in the *C. annuum* CMS lines. The amplification profiles of those markers differed among the fertile *C. annuum* accessions, except for the Korean landraces Yuwol and Chilsung. In the other *Capsicum* species, seven markers were amplified in *C. chinense* (Habanero) and/or *C. frutescens* (AC08-026), while *C. baccatum* (AC08-062) and *C. chacoense* (AC08-075) did not show clear amplification of any of the 20 markers (**Figure 2**).

We applied the 20 SCAR markers to a total of 144 accessions of *C. annuum*, *C. chinense*, *C. frutescens*, and *C. baccatum*, most of which were included in the core collection developed by Lee et al. (2016). Most of the markers were clearly either present or absent each of the 96 *C. annuum* accessions. We did, however, detect very weak DNA bands, which may represent the existence of target DNA at substoichiometric levels (**Supplementary Table S2; Supplementary Figure S3**). We did not distinguish those weak bands from absence of amplification because 1) many mtDNA regions that had been regarded as absent in specific germplasms have been shown to actually exist in those germplasms at very low copy number (Arrieta-Montiel et al., 2001) and 2) success or failure in the detection of very weak DNA amplicons is dependent on experimental conditions because of the extremely low copy number of the target DNA regions.

We classified the mitotypes of the 96 *C. annuum* accessions into 15 groups based on the amplification profiles of the SCAR markers (**Table 1**, **Supplementary Table S2**). Groups 1, 4, 8, 12, and 13 contained 13, 23, 39, 4, and 3 accessions, respectively, while the other groups contained one or two accessions each. The mitotypes of Jeju Jaerae and FS4401 were the same as those of groups 8 and 12, respectively. The markers designed from the Jeju Jaerae sequence showed a similar genotyping pattern in most of the accessions. In contrast, markers 1 and 2, which were designed from the FS4401 sequence, could classify the accessions into four large groups (group 1–3, group 4–7, group 8–11 and 13–15, and group 12; **Table 1**).

**Table 1:** Classification of germplasms using mitotype markers.

Classification using 20 markers	Classification using MCM1	Mitotyping markers																				Number of germplasms
		1^z^	2	3	4	5	6	7	8	9	10	11	12	13	14	15	16	17	18	19	20	
Group 1	Type 1	Y^y^	N^x^	N	N	N	N	N	N	N	N	N	N	N	N	N	N	N	N	N	N	13
Group 2		Y	N	N	N	N	N	N	N	N	N	N	N	N	N	N	N	N	Y	N	N	2
Group 3		Y	N	N	N	N	N	N	N	N	Y	Y	Y	N	Y	Y	N	N	N	N	N	1
Group 4	Type 2	N	Y	N	N	Y	Y	Y	Y	N	N	N	N	Y	Y	Y	N	N	N			23
Group 5		N	Y	N	N	Y	Y	Y	Y	N	N	N	N	Y	Y	Y	N	N	N		N	1
Group 6		N	Y	N	N	Y	Y	Y	Y	N	Y	Y	Y	Y	Y	Y	N	N	N			1
Group 7		N	Y	N	N	Y	Y	Y	Y	Y	Y	Y	Y	Y	Y	Y	N	N	N			1
Group 8	Type 3	N	N	N	N	Y	Y	Y	Y	Y	N	N	N	Y	Y	Y	N	N	N			39
Group 9		N	N	N	N	Y	Y	Y	N	N	N	N	N	Y	Y	Y	N	N	N	Y		2
Group 10		N	N	N	N	Y	Y	Y	Y	N	N	N	N	Y	Y	Y	N	N	N			2
Group 11		N	N	N	N	Y	Y	Y	Y	Y	N	N	N	Y	Y	Y	N	N	N	Y	N	2
Group 12	Type 4	Y	Y	Y	Y	N	N	N	N	N	Y	Y	Y	N	N	N	Y	Y		N	N	4
Group 13	Type 5	N	N	N	N	N	N	Y	N	N	N	N	N	N	Y	Y	N	N	N	N	N	3
Group 14		N	N	N	N	Y	N	Y	Y	N	N	N	N	Y	Y	Y	N	N	N			1
Group 15		N	N	N	N	N	N	N	N	N	N	N	N	N	N	N	N	N	N	N	N	1

^z^Markers with underlines were designed from FS4401; others were designed from Jeju Jaerae.

^y^Y: amplifications with high yield.

^x^N: amplifications with low yield or no amplification.

Sequencing of selected amplicons showed that the amplicon sequences were the same or highly similar (>99%) to the original sequences from which markers were designed. The internal sequences of the marker 2 amplicons from four accessions in group 1 matched perfectly to the sequence of the CMS candidate gene *orf507*. Another CMS candidate gene, *Ψatp6-2*, was detected only in the accessions in group 12 (data not shown).

The SCAR markers produced many DNA bands in other *Capsicum* species that were weaker than the bands of the saturated amplification but stronger than the typical weak bands in the *C. annuum* accessions (**Supplementary Table S3; Supplementary Figure S4**). We determined that our SCAR markers are not suitable for precise mitotyping in *Capsicum* species other than *C. annuum* because of the ambiguous amplification results. Thus, SCAR markers seemed to be useful for classification of mitotypes only for *C. annuum* accessions.

### Development of a Multiplex PCR System for Efficient Classification of Mitotypes in *C. annuum*

We designed a multiplex PCR system using markers that could efficiently classify *C. annuum* mitotypes into large groups. Among the groups based on the amplification patterns of the 20 SCAR markers, groups 1, 4, 8, 12, and 13 were the major groups containing at least three accessions (**Table 1**). The groups could be differentiated using only four markers, which were marker 1; marker 2; any one of markers 3–4, 10–12, and 16–18; and any one of markers 5–6 and 8–9. We selected markers 3 and 6 because they resulted in the fewest weak amplifications among the respective candidates. The application of the selected markers to three individuals per accession resulted in consistent genotyping results showing stability of markers in genotyping. We designed new primer pairs (marker 3-S and 6-S) for markers 3 and 6 to ensure that there would be enough difference in size between the amplicons.

**Figure 3:**
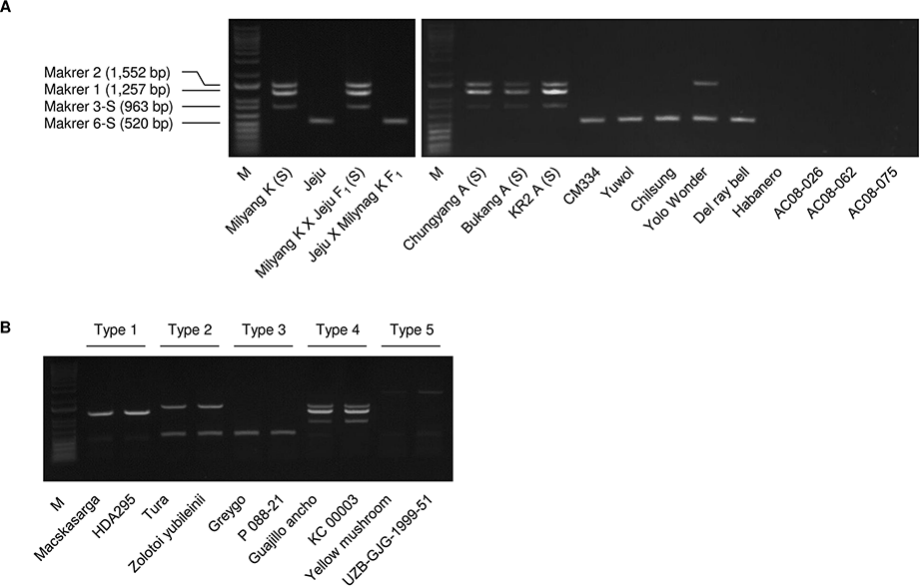
Amplification patterns in multiplex PCR using the MCM1 marker. **(A)** Application of MCM1 to a panel of *Capsicum* accessions including CMS lines. Jeju Jaerae is abbreviated as “Jeju.” **(B)** Amplification pattern for the five *C. annuum* mitotypes detected. M refers to a size marker.

**Figure 4:**
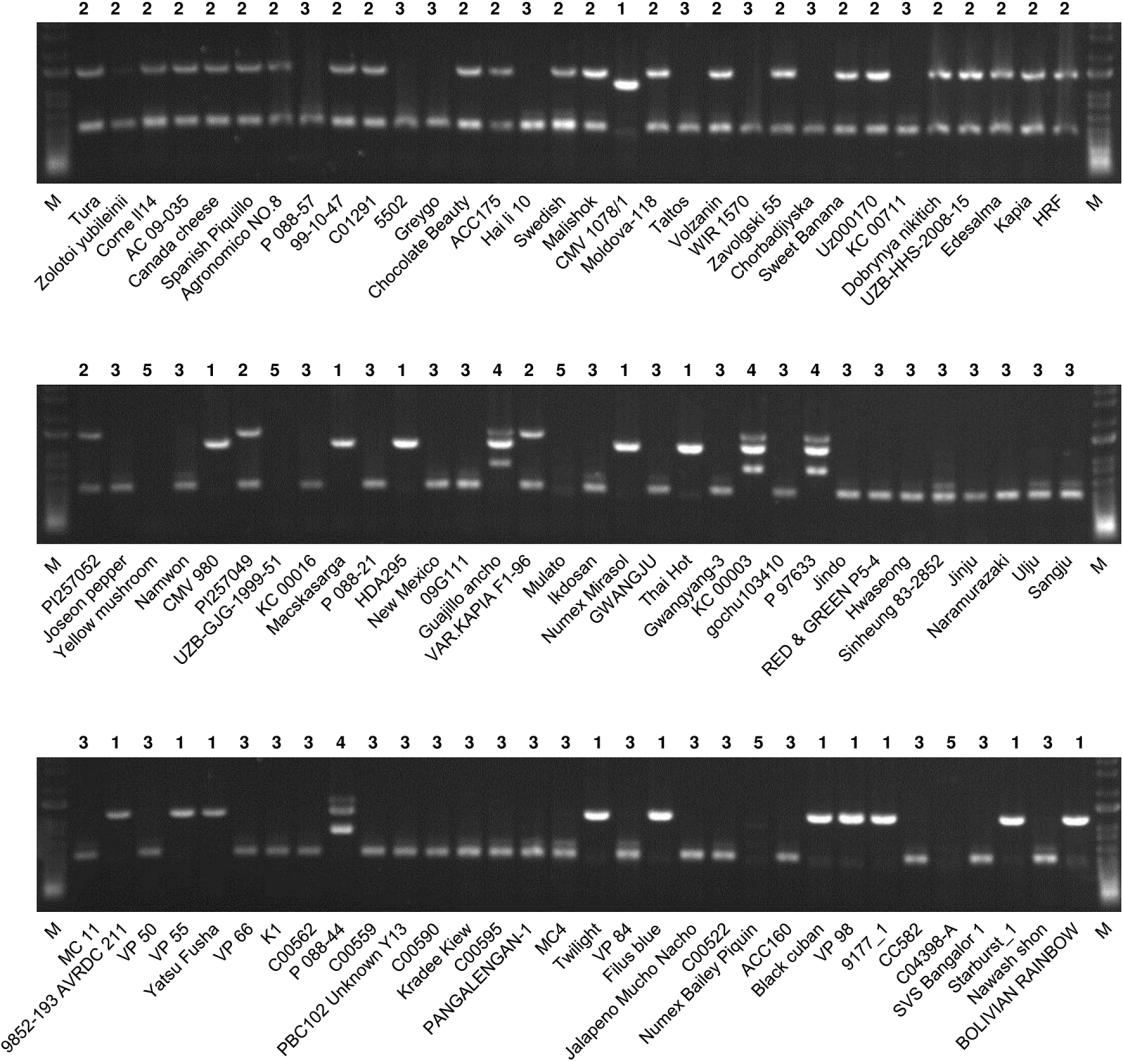
Application of MCM1 to 96 accessions of *C. annuum*. The mitotypes of the accessions defined by this marker are written above each lane. M refers to a size marker.

The multiplex PCR using markers 1, 2, 3-S, and 6-S (**Supplementary Table S4**) resulted in clear amplification patterns in application to *C.annuum* accessions (**Figures 3** and **4**; **Supplementary Table S2**). We named the multiplex PCR marker MCM1. The application of MCM1 resulted in five distinctive amplification patterns (**Figure 3B**) corresponding to five different mitotypes, which we refer to as “MCM-type 1” to “MCM-type 5” for convenience. MCM-type 3 was the most common mitotype, appearing in Jeju Jaerae and in 45 other *C. annuum* accessions. MCM-type 4 was the mitotype of CMS accessions used in our preliminary application of the individual markers (**Figures 2** and **3**). MCM-type 4 appeared in four of the 96 additional *C. annuum* accessions. MCM-types 1, 2, and 5 appeared in 16, 26, and 5 *C. annuum* accessions, respectively (**Figure 4**; **Supplementary Table S2**). The PIC value of MCM1 was 0.67, which was significantly higher than that of any single SCAR marker (0.08–0.49; **Supplementary Table S7**).

### Characteristics of the Classification Pattern According to Mitotypes Among the *C. annuum* Accessions

**Figure 5:**
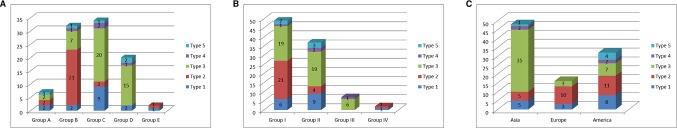
Relationship of the mitotypes determined by MCM1 with phylogenetic groups determined by nuclear SNP markers and geographic origins of 96 *C. annuum* accessions. **(A)** Relationship of mitotypes with groups classified by population-structure analysis of 3,821 *Capsicum* accessions based on nuclear SNP markers (Lee et al., 2016). **(B)** Relationship of mitotypes with phylogenetic groups classified by application of nuclear SNP markers to 240 *Capsicum* accessions in a core collection (Lee et al., 2016). **(C)** Relationship of mitotypes with geographic origins of the accessions.

We compared the classification of the *C. annuum* accessions based on mitotypes with those based on nuclear SNP markers and geographic origins (Lee et al., 2016; **Figure 5**). Lee et al. (2016) constructed a core collection (240 *Capsicum* accessions) by analysis based on marker application and phenotyping from a total of 3,821 *Capsicum* accessions in which population structure analysis was performed using 48 SNP markers. The *C. annuum* accessions used in our research were grouped into five clusters (groups A–E) according to that analysis. The MCM-type 2 accessions were mainly included in group B, while the MCM-type 3 accessions were largely distributed in groups C and D. The MCM-type 1 accessions were spread among all of the groups, but most were in group C (**Figure 5A**).

Lee et al. (2016) previously used 48 nuclear SNP markers to classify the 96 *C. annuum* accessions in their core collection mostly into three groups (groups I–III) based on phylogenetic analysis. Most of MCM-type 2 accessions were in group I. Meanwhile, most of the type 1 and 3 accessions were in groups I and II (**Figure 5B**). In terms of geographic origins, most of the accessions from Asia and Europe were MCM-type 3 (72.9%) and MCM-type 2 (62.5%), respectively. The accessions from North and South America included all of the MCM-types (**Figure 5C**).

**Figure 6:**
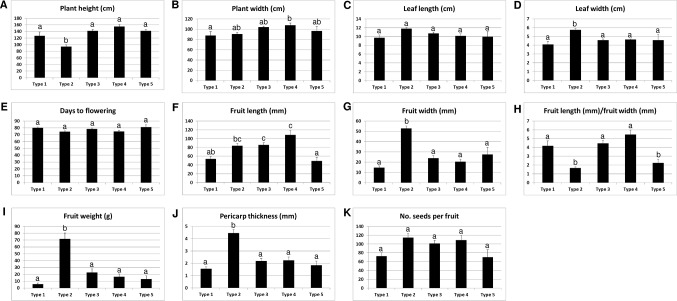
Relationship of the mitotypes determined by MCM1 with 11 phenotypic characteristics in 96 *C. annuum* accessions. Mean values are indicated with ± standard error, shown as error bars. Groups regarded to be different from each other in Duncan’s multiple range tests at the *P* < 0.05 level are indicated by different letters above the graphs.

We analyzed the accessions of the five mitotypes for 11 characteristics (10 traits and the “length-to-width ratio of fruits”) related to plant architecture, flowering, and fruit morphology (**Figure 6**). Multiple comparisons of the mean values revealed significant differences among the mitotypes in eight characteristics. Notably, the MCM-type 2 accessions were clearly differentiated from all of the other mitotypes in plant height, leaf length, fruit width, fruit weight, and pericarp thickness (**Figures 6A, C, G, I, J**). The MCM-type 2 accessions had shorter plant height, had shorter leaves, and bore heavier and wider fruits with thicker pericarp. The MCM-type 3 accessions, the largest mitotype group, had much narrower and lighter fruits than the MCM-type 2 accessions (**Figures 6G, I**). The MCM-type 1 accessions were differentiated from the MCM-type 3 accessions by a shorter fruit length, although they were not different than the MCM-type 3 accessions in terms of fruit width and pericarp thickness (**Figures 6F, G, I**). MCM-type 5 accessions were similar to the MCM-type 1 accessions in average fruit length and pericarp thickness, but they had a lower length-to-width ratio (**Figure 6H**). MCM-type 4 included only four accessions with relatively high variation among them, so an investigation of a larger number of accessions of that mitotype is required.

We analyzed the same phenotypic characteristics among the groups defined on the basis of the 48 nuclear SNPs. There were significant differences among the groups in seven of the 11 characteristics (**Supplementary Figure S5**). The group I accessions, which include the largest number of MCM-type 2 accessions, had heavier and wider fruits with thicker pericarp than groups II and III (**Supplementary Figures S5D, F, G**). However, the distinctiveness of those characteristics was more pronounced in MCM-type 2 accessions than in group I. For example, the average values for fruit width, fruit weight, and pericarp thickness were significantly higher (*P* < 0.05) for MCM-type 2 (52.9 mm, 71.9 g, and 4.4 mm, respectively) than for group I (38.6 mm, 47.8 g, and 3.3, respectively; **Figure 6**; **Supplementary Figure S5**).

### Analysis of the Relationships of the Mitotypes Based on SNPs or InDels

Because SCAR markers for structural variations reflect not only the presence or absence of specific structures but also increases or decreases of the copy number of subgenomic molecules, SCAR markers may not be adequate to determine the order of events in evolutionary processes. Therefore, we developed two mtDNA markers based on SNPs or InDels in mtDNA and applied them to a total of 88 accessions from four *Capsicum* species (**Supplementary Table S8**). The first marker, mt-227, grouped the accessions (except for the *C. baccatum* accessions in which Sanger sequencing detected multiple sequences) into three types (**Table 2**; **Supplementary Figure S1A**). The mt-227 type 1 included all MCM-type 1, 4, and 5 *C. annuum* accessions, except for one MCM-type 5 accession (**Table 2**; **Supplementary Table S8**). All, but one, of the mt-227 type 2 accessions were MCM-type 2 or MCM-type 3 *C. annuum* accessions. Finally, mt-227 type 3 included all *C. frutescens* accessions and most *C. chinense* accessions, although three and two *C. chinense* accessions were mt-227 type 2 and type 1, respectively. All four CMS lines were mt-227 type 1, as were most of the MCM-type 1, 4, and 5 *C. annuum* accessions. Sequence alignment among the three mt-227 types showed that mt-227 type 3 is more similar to mt-227 type 1 than to mt-227 type 2 (**Supplementary Figure S1A**).

**Table 2:** Classification of germplasms using markers based on mtDNA sequences.

Species	Classification using MCM1	Classification using 20 SCAR markers	mtDNA markers	
			mt-227	mt-139
*C. annuum* (40)^z^	Type 1 (8)	Group 1 (5)	Type 1 (8)	N^y^ (8)
		Group 2 (2)		
		Group 3 (1)		
	Type 2 (8)	Group 4 (5)	Type 2 (19)	Y (19)
		Group 5 (1)		
		Group 6 (1)		
		Group 7 (1)		
	Type 3 (11)	Group 8 (5)		
		Group 9 (2)		
		Group 10 (2)		
		Group 11 (2)		
	Type 4 (8)^x^	Group 12 (4)	Type 1 (8)	N (8)
	Type 5 (5)	Group 13 (3)	Type 1 (3)	N (3)
		Group 14 (1)	Type 2 (1)	Y (1)
		Group 15 (1)	Type 1 (1)	N (1)
*C. frutescens* (16)	–^w^	–	Type 3 (16)	N (16)
*C. chinense* (16)	–	–	Type 3 (11)	N (11)
			Type 2 (3)	Y (2)
				N (1)
			Type 1 (2)	N (2)
*C. baccatum* (16)	–	–	ND^v^ (16)	N (16)

^z^Number in parenthesis refers to the number of accessions.

^y^Y and N refer to presence or absence of the amplicon for mt-139.

^xT^his group contained four breeding lines with CMS cytoplasm.

^w^Groups in which mitotypes could not be clearly determined using MCM1 are indicated by “-.”

^v^ND, inability to determine marker genotype due to the presence of multiple amplicons.

The mt-139 marker is a SCAR marker based on an InDel. That marker classified the accessions into two types according to the presence or absence of amplicons (**Table 2**; **Supplementary Table S8**). Mt-139 was amplified in all mt-227 type 2 accessions but not in the other accessions (including the CMS lines), except for one *C. annuum* and two *C. chinense* accessions that were also exceptional in their classification by mt-227. Thus, genotyping by mt-139 was highly consistent with that by mt-227 (**Table 2**).

A SCAR marker based on a plastid sequence (PepRpl; Jo et al., 2011) produced a different classification pattern. That marker was amplified in all MCM-type 2 and 4 *C. annuum* accessions as well in as many of the MCM-type 3 and 5 accessions. The PepRpl marker grouped all four CMS lines together with the MCM-type 2 accessions, whereas the two mtDNA markers did not (**Supplementary Table S8**).

### Screening of Candidate CMS-Associated Genes

**Figure 7:**
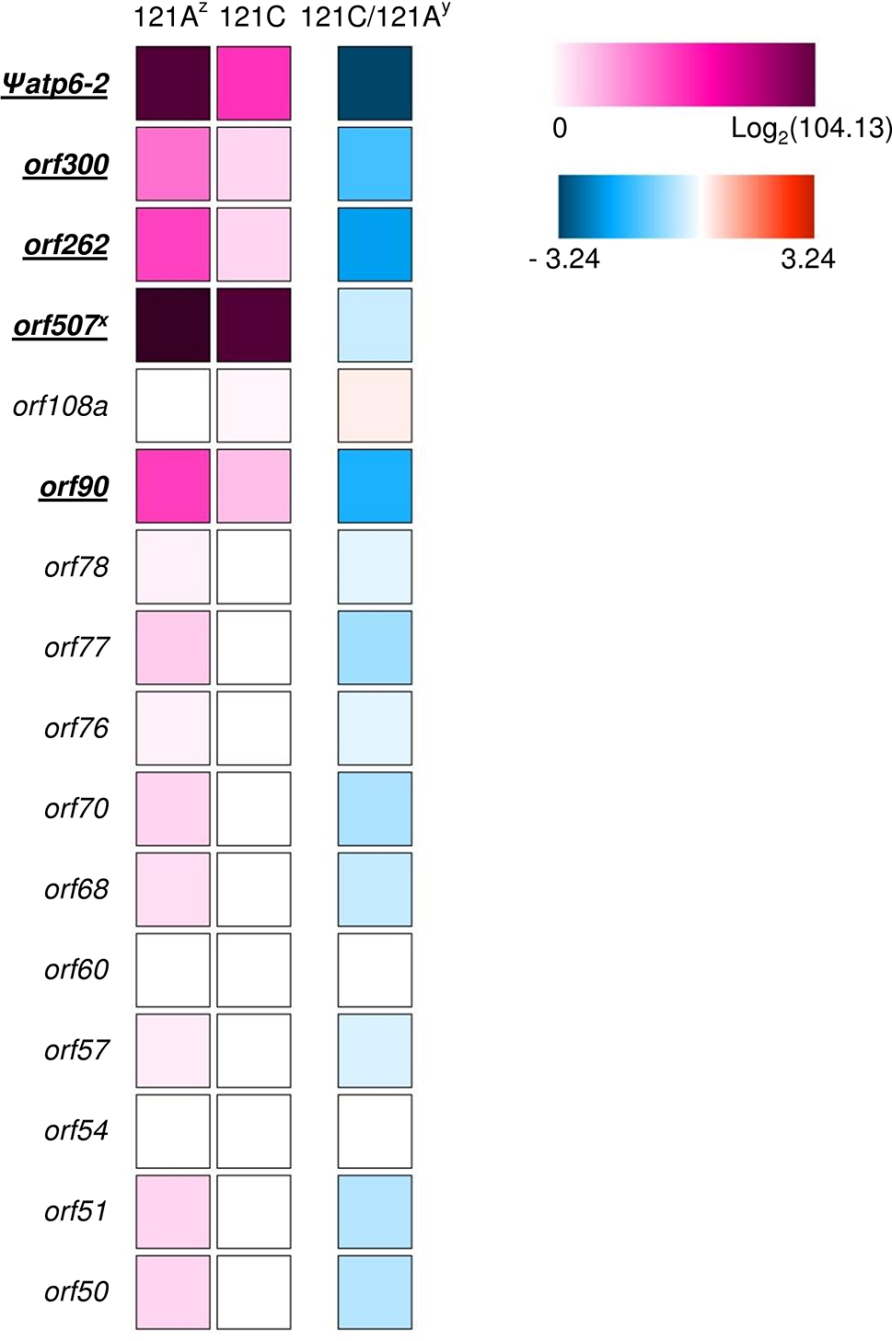
Heat map of RPKM of transcriptome sequence reads from a CMS line (121A) and its near-isogenic restorer line (121C) showing the candidate *orf*s for CMS-associated genes. The *orf*s showing RPKM higher than three are underlined. ^z^ Logarithm of the RPKM + 1 value for 121A and 121C are presented using colored squares below “121A” and “121C,” respectively. ^y^ Logarithm of the ratio between the RPKM + 1 value of two lines is presented using colored squares below “121C/121A.” ^y^ Except for *orf507*, which was named according to its length in nucleotides in a previous study, the numbers in the names of other *orf*s represent the amino acid length of the protein products.

To select reasonable CMS candidate genes, we screened candidate *orf*s, analyzed *orf* transcription, and investigated the distribution of *orf*s in *Capsicum* accessions. We screened the *orf*s in the FS4401 mtDNA against those in the Jeju Jaerae mtDNA to determine which ones are specific to FS4401 (the CMS line). Then, we selected the FS4401-specific *orf*s that are predicted to contain transmembrane domains because most of the CMS-associated genes that have been cloned in other crops contain transmembrane domain(s) (Kubo et al., 2011). That process resulted in the selection of a total of 16 *orf*s (**Supplementary Table S6**). Using data from a previous study that mapped transcriptome reads from a CMS line and its near-isogenic restorer line (Liu et al., 2013), we found that only five of the selected *orf*s, including the candidate CMS genes *orf507* and *Ψatp6-2* (Kim and Kim, 2006; Kim et al., 2007b), were transcribed with an RPKM value higher than three (**Figure 7**; **Supplementary Table S5**). The expression of two of *orf*s (*Ψatp6-2* and *orf262*) was more than 10 times greater in the CMS line than in the restorer line. The expression of two other *orf*s (*orf300* and *orf90*) was about six times greater in the CMS line than in restorer line, while that of *orf507* was similar between the two lines (**Figure 7**; **Supplementary Table S5**). For validation, we performed qRT-PCR analysis for the five *orf*s in our CMS and restored materials. The efficiency of qRT-PCR for each *orf* or genes ranged from 92.3 to 100.1%. In the qRT-PCR analysis, significantly decreased expression of four *orf*s (*Ψatp6-2*, *orf262*, *orf300*, and *orf90*) except for *orf507* were detected when the expression levels were normalized against a mitochondrial rRNA gene, *rrn26*, although the differences between the CMS and restored materials were smaller compared to those in transcriptome analysis. Another mitochondrial rRNA gene, *rrn18*, did not show significant difference in transcription level between the CMS and restored plants in this analysis (**Supplementary Figure S6**). Investigation of the five *orf*s in 144 *C. annuum* accessions and four CMS lines showed that *Ψatp6-2*, *orf262*, and *orf90* were specific to MCM-type 4 and the CMS accessions (**Supplementary Table S9**). On the other hand, *orf300* and *orf507 *were present in all MCM-type 1 and 2 accessions, respectively. In other *Capsicum* species, the “Toko Subur” accession of *C. frutescens* contained *Ψatp6-2*, *orf262*, and *orf300*. In addition, three *C. chinense* accessions contained *orf300*.

## Discussion

### Characteristics of Markers Based on Structural Variation in mtDNA in Pepper

Extensive structural variation due to DNA rearrangements and substoichiometric shifts is a unique feature that distinguishes plant mitochondrial DNA from that of animals. Such variation has been observed even between accessions within the same plant species (Kim et al., 2007b; Davila et al., 2011; Kubo et al., 2011). DNA structural variation may be especially useful for intraspecific analysis based on mitotypes in plants because the evolutionary rate of DNA structural change is higher than that of nucleotide substitution in mitochondrial genes, although mitochondrial DNA structural markers and mitochondrial SNP markers result in classifications that are not contradictory to each other (Kress et al., 2005; Davila et al., 2011).

We designed markers based on three aspects of structural variation to increase their reliability. First, we excluded rearrangement junctions involving small repeated sequences because internal repeat sequences might produce artificial amplicons (Alverson et al., 2011). Second, we screened and excluded possible Numt sequences using BLAST analysis and application to progenies from reciprocal crosses because large portions of mitochondrial DNA sequences can become integrated into the nuclear genome. For example, *Vitis vinifera*, which has a mitochondrial genome 773 kb in size, contains a total of 1,603 kb of Numt sequences (Ko and Kim, 2016). We cannot rule out the possibility that our selected sequences might have been integrated into the nuclear genome in *Capsicum* accessions other than the three lines (CM334, Jeju Jaerae, Milyang K). However, at least, any of marker amplicons were not estimated as that of Numts in our sequencing analysis on amplicons from several accessions. The existence of subgenomic mtDNA molecules at substoichiometric levels can also be a potential drawback in the application of mtDNA markers. Arrieta-Montiel et al. (2001) detected at least very small amounts of amplicons from a CMS-associated gene in all of the *P. vulgaris* accessions that they tested. We reduced the risk of incorrect scoring due to weak amplification by selecting SCAR markers that showed clear amplification and by using a multiplex PCR system that restricts the amount of each amplicon. We did, however, detect low to moderate levels of amplification in accessions from *Capsicum* species other than *C. annuum*. That may be because we used *C. annuum* accessions to design and optimize the markers, and the evolutionary states and contents of mtDNA can be very different even between species within a genus (Tang et al., 2017). Our results indicate that our mtDNA markers are useful for the subdivision of accessions within *C. annuum* when precise mitotype analysis is the aim.

### Utility of the mtDNA Markers in the Classification of *C. annuum* Accessions

Although mtDNA markers have been developed previously in pepper, they mainly focused on distinction between CMS and normal cytoplasm and not on the classification of diverse mitotypes (Kim and Kim, 2005; Kim and Kim, 2006; Gulyas et al., 2010). A recent study developed simple sequence repeat (SSR) markers based on many positions in the mitochondrial genome; however, the authors did not use the markers to classify different accessions (Cheng et al., 2016). We not only developed markers based on structural variations dispersed throughout the mitochondrial genome but also used the markers to classify diverse *Capsicum* accessions and analyzed the relationship between mitotypes and phenotypes, geographic origins, and nuclear marker-based phylogenetic groups.

Our multiplex PCR marker system containing four SCAR markers only detected five different mitotypes, although a total of 16 mitotypes could exist theoretically. Possible pairs of any two of the four markers, except for the pair of marker 1 and marker 6, did not result in simultaneous amplifications in any *C. annuum* accessions except for those with the MCM-type 4 mitotype. That result implies that new DNA rearrangements unique to each mitotype have occurred during evolution or that specific substoichiometric states of subgenomic molecules that arose once have been maintained. Therefore, we can infer that mitotypes determined by mitochondrial DNA structure can be a stable indicator for the study of *C. annuum* diversity.

The mitotypes of the *C. annuum* accessions were related to the origins and morphological characteristics of the accessions. Although previous studies have reported close relationships between fruit characteristics and nuclear marker genotypes (Hill et al., 2013; Nicolaï et al., 2013; Lee et al., 2016), such relationships have never been reported with mitotypes in peppers. Because most of the accessions used in our study were a subset of those used in a previous study (Lee et al., 2016), we could directly compare our results to those based on the nuclear DNA markers of the previous study. The accessions of each mitotype were spread among the nuclear DNA marker-based phylogenetic groups without a clear pattern except for the MCM-type 2 accessions, which were mainly included in group I. The accessions in group I, like the MCM-type 2 accessions, were characterized by large fruits with a thick pericarp, but the distinctiveness of those characteristics was much more pronounced in the classification by mtDNA markers. Therefore, our mtDNA markers may provide new tools for germplasm classification that are different from and supplementary to nuclear DNA markers.

### The Relationship Between Mitotypes and Domestication

The mitotypes differed significantly from one another especially in many fruit characteristics. The MCM-type 1 accessions bore the lightest fruits and represented the largest portion of *C. annuum* accessions with erect-type fruits (eight out of 12 accessions with erect-type fruits were MCM-type 1; data not shown). On the other hand, the MCM-type 2 and 3 accessions had the highest average fruit weight. The MCM-type 2 accessions largely included bell-type peppers that are mostly from European countries, while the MCM-type 3 accessions mostly bore long and narrow fruits, which are popular in Asian countries.

**Figure 8:**
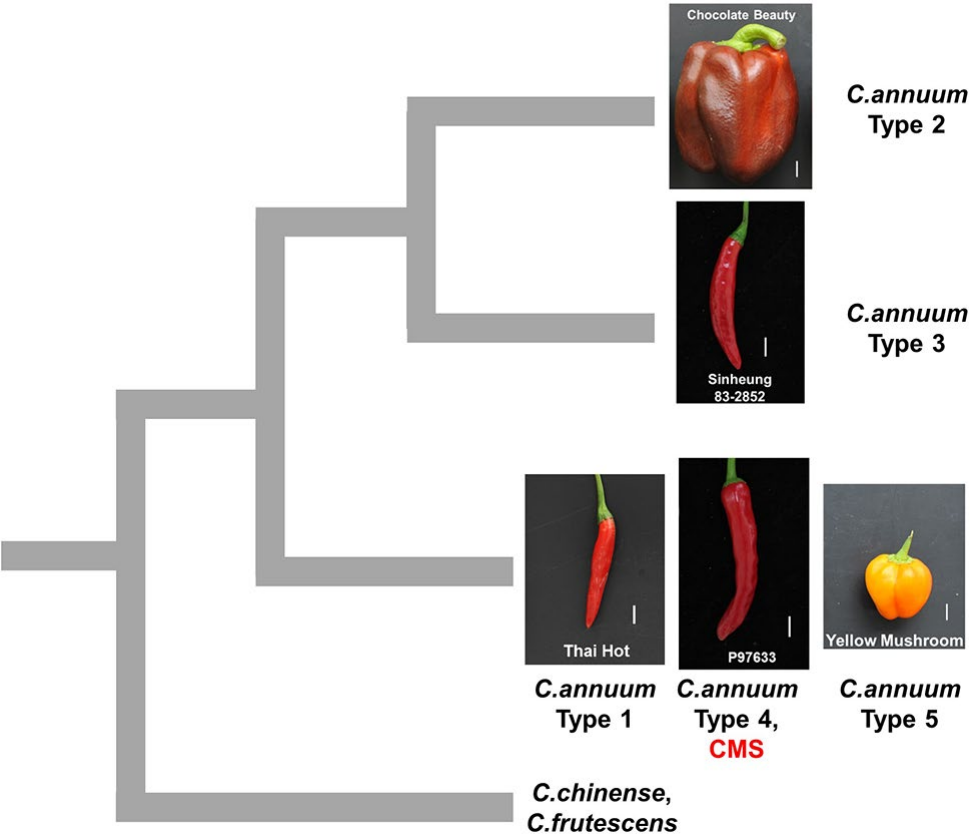
Schematic diagram showing hypothetical relationships among pepper groups classified by mtDNA markers. The mitotypes classified by MCM1 are written as types 1 to 5. The pictures are fruits of accessions that are close to the average in each group in terms of fruit length and width. The scale bars represent 1 cm.

The genotypes of the MCM-type 2 and 3 accessions as determined by the SNP or InDel-based markers were clearly distinctive from those of all the other *Capsicum* accessions, suggesting that they are diverged from the other accessions (**Figure 8**). The grouping of pepper accessions that bear heavier fruits might be related to domestication process since domestication of peppers is thought to have involved improvement in the weight and size of fruits following the selection of peppers that bear nondeciduous and pendant fruits in contrast to the wild progenitor peppers that bear small, erect, and deciduous fruits (Bosland and Votava, 2000; Paran and van der Knaap, 2007). It is notable that the classification of those accessions not only involved stepwise small-scale sequence variation (SNPs and small InDels) in genomic (Lee et al., 2016) and mitochondrial DNA but also was accompanied by extensive structural variation in the mitochondrial genome. That suggests either mtDNA structural variations affected characteristics related to selection during domestication or diversification or they were accompanied by agronomic characteristics without direct influence on them.

For the former possibility, extensive change on mitochondrial genome can influence the nucleocytoplasmic interactions, which in turn is related to agronomic characteristics (Jacoby et al., 2012; Greiner and Bock, 2013). Since majority of mitochondrial proteins (93–99%) are coded by nuclear genome (Woodson and Chory, 2008) and mutiprotein complexes in mitochondria are formed by combination of subunits being coded by nuclear and mitochondrial genomes, these genomes coevolve to optimize compatibility between them (Moison et al., 2010; Ng et al., 2014). Thus, extensive change on mtDNA may result in unique combination of nuclear and mitochondrial genomes as the outcomes of coevolution, which can affect performance of biological processes in mitochondria. This can also influence chloroplast function due to extensive interactions between mitochondria and chloroplast in energy, metabolism, and redox status (Ng et al., 2014). In several studies, perturbation of respiration and carbon metabolism in mitochondria affected the performance of photosynthetic carbon assimilation in chloroplast (Sabar et al., 2000; Nunes-Nesi et al., 2014; Priault et al., 2006) that has been known to be closely related to fruit development (Aroujo et al., 2014; Azzi et al., 2015). In addition, substitution or modification of mitochondrial genome can affect expression of nuclear genes *via* retrograde signaling (Ng et al., 2014). For example, upregulation of a large number of genes related to cellular protein metabolism was detected in a citrus cybrid in which mitochondria was substituted by somatic hybridization (Bassene et al., 2011). The expression of several genes involved in the Calvin cycle and light reactions was also changed, implying cross-talk between mitochondria and chloroplast. Except for possible changes in regulatory or protein-coding sequences of known genes, the *orf*s that existed and were expressed specifically in accessions with certain mitotypes in our analysis also may affect these interactions or signaling. For example, *orf507*, which was specifically amplified in MCM-type 2 and 4 accessions, had been shown to interact with a subunit of mitochondrial ATP synthase that is coded by a nuclear gene (Li et al., 2013).

Regarding the latter possibility, diversification or domestication might have involved wide crosses that induced incompatibility between nuclear and mitochondrial genomes and thus led to rearrangements of the mitochondrial genome (Hanson and Bentolila, 2004). In addition, recent studies have shown that suppression of MutS HOMOLOG1 (MSH1), which results in illegitimate recombination in mtDNA, also could induce epigenetic developmental reprogramming triggered by plastid signal (Virdi et al., 2015). Cross of lines derived from mutants with different wild-type lines leads to inheritable improvement of agronomic characteristics in sorghum (Santamaria et al., 2014), tomato (Yang et al., 2015), and soybean (Raju et al., 2018).

Further studies including reciprocal crosses between accessions with different mitotypes are needed to examine the possibility that evolution of the mitochondrial genome is related to characteristics associated with domestication.

### Elucidation of the Origin of CMS and CMS-Associated Genes

In pepper, three independent sources of CMS cytoplasms have been reported: two from *C. annuum* accessions by spontaneous occurrence (Peterson, 1958) and intraspecific crosses (Shifriss and Frankel, 1971), respectively, and one from *C. frutescence* by an interspecific cross between *C. frutescens* and *C. annuum* (Yu, 1990). Test crosses with maintainer and restorer lines showed that those lines have the same potential for sterilization and fertility restoration (Shifriss, 1997). All CMS lines used in our study showed the same genotypic profile as the type 4 *C. annuum* accessions for all the markers. Analysis by mt-227, the marker based on mtDNA SNPs and InDels, indicated that CMS and MCM-type 4 accessions are grouped with type 1 and 5 *C. annuum* accessions, whereas they are distinguished from MCM-type 2 and 3 *C. annuum* accessions and most of the accessions of other *Capsicum* species. That implies that the CMS cytoplasms in our study likely originated from the progenitor-type *C. annuum* germplasm rather than the recently diverged germplasm with the heavier fruits (**Figure 8**).

Application of another marker, mt-139, confirmed the separation of the CMS and MCM-type 4 accessions from the MCM-type 2 and 3 accessions. That result is contradictory to our previous report in which we concluded on the basis of plastid-based markers that the CMS cytoplasm was from a more domesticated germplasm (Jo et al., 2011). However, the plastid-based marker produced results for the CMS and MCM-type 4 *C. annuum* accessions that were inconsistent with the results produced by the mtDNA markers, implying that the conclusion in previous study was not correct. The collapse of coinheritance of organellar genomes might be due to rare biparental inheritance of plastid genomes (Barnard-Kubow et al., 2017).

The subdivision of mitotypes enabled us to screen for CMS candidate genes with higher resolution. The presence of *orf507*, a candidate CMS gene, in accessions with normal cytoplasm has been reported previously (Jo et al., 2009; Jo et al., 2011; Ji et al., 2014). Our analysis supported and advanced those findings because *orf507* was a crucial criterion to define the type 2 accessions, which included many accessions with specific fruit characteristics. In addition, we showed that Yolo Wonder, an accession with normal cytoplasm (Wang et al., 2004), contains the complete *orf507* sequence. Therefore, it may be necessary to reconsider the status of *orf507* as a CMS candidate gene after its expression is tested in accessions with normal cytoplasm in further studies. Among the other *orf*s predicted to encode proteins with a transmembrane domain, *Ψatp6-2*, *orf262*, and *orf90* were highly specific to the CMS and type 4 accessions of *C. annuum*. They also showed decreased transcription in the presence of *Rf*. Decrease of CMS gene transcripts in the presence of *Rf* has been shown in many crops, although other mechanisms, such as translational or posttranslational regulations, also have been reported for fertility restoration (e.g., translational suppression of *orf138* by *Rfo* in *Brassica* and radish, structural change of preSATP6 by *BvORF20* in sugar beet; Kim and Zhang, 2017). Recently, Wang et al. (2019) proposed CMS candidate genes based on comparative analysis on mtDNA sequences and gene expression levels between a CMS and a normal pepper line. They suggested *orf300a* and *orf314a*, which correspond to *orf300* and *Ψatp6-2* in our study, respectively, as strong candidates. Although the corelationship of *orf300a* with CMS was shown in many breeding lines, diversity of lines was not represented. In addition, candidates were screened for *orf*s that code for proteins 100 amino acids or longer in length, although *orf*s that code for proteins shorter than 100 amino acids in length have been reported as CMS-associated genes in several crops (e.g., *orf79* in rice Boro cytoplasm and *orf77* in maize S cytoplasm; Hanson and Bentolila, 2004). In our study, pepper accessions with classified mitotypes were additionally used for CMS candidate gene selection among CMS cytoplasm-specific *orf*s that coded for proteins 50 amino acids or longer in length. Considering lack of efficient transformation system for direct validation of gene function in pepper (Van Eck, 2018), strict selection of candidate genes based on comparison of complete mitochondrial genome sequences and classification of mitotypes in present study will contribute to molecular breeding as well as studies on CMS mechanism.

## Author Contributions

YJ participated in design of the study, marker development, and data analysis. H-YL performed germplasm collection, DNA extraction, and development of phenotype database. N-YR involved in germplasm collection and phenotyping. SK and J-BK supported genotyping analysis. B-CK participated in discussion and revision of manuscript. S-YK managed the project that supported this research.

## Funding

This work was carried out with the support of the National Research Foundation of Korea (NRF) grant (Project number 2017M2A2A6A05018543) funded by the Korean government and Cooperative Research Program for Agriculture Science & Technology Development (Project number PJ01322901) funded by Rural Development Administration, Republic of Korea.

## Conflict of Interest

The authors declare that the research was conducted in the absence of any commercial or financial relationships that could be construed as a potential conflict of interest.
