# Supplementary material for: Mitotypes Based on Structural Variation of Mitochondrial Genomes Imply Relationships With Morphological Phenotypes and Cytoplasmic Male Sterility in Peppers
Source: Front Plant Sci. 2019 Oct 24;10:1343. doi: 10.3389/fpls.2019.01343 (PMC6822277; doi:10.3389/fpls.2019.01343)
Supplement: Supplementary file 1 [file DataSheet_1.pdf]

Supplementary Material

1 Supplementary Figures and Tables

1.1 Supplementary Figures

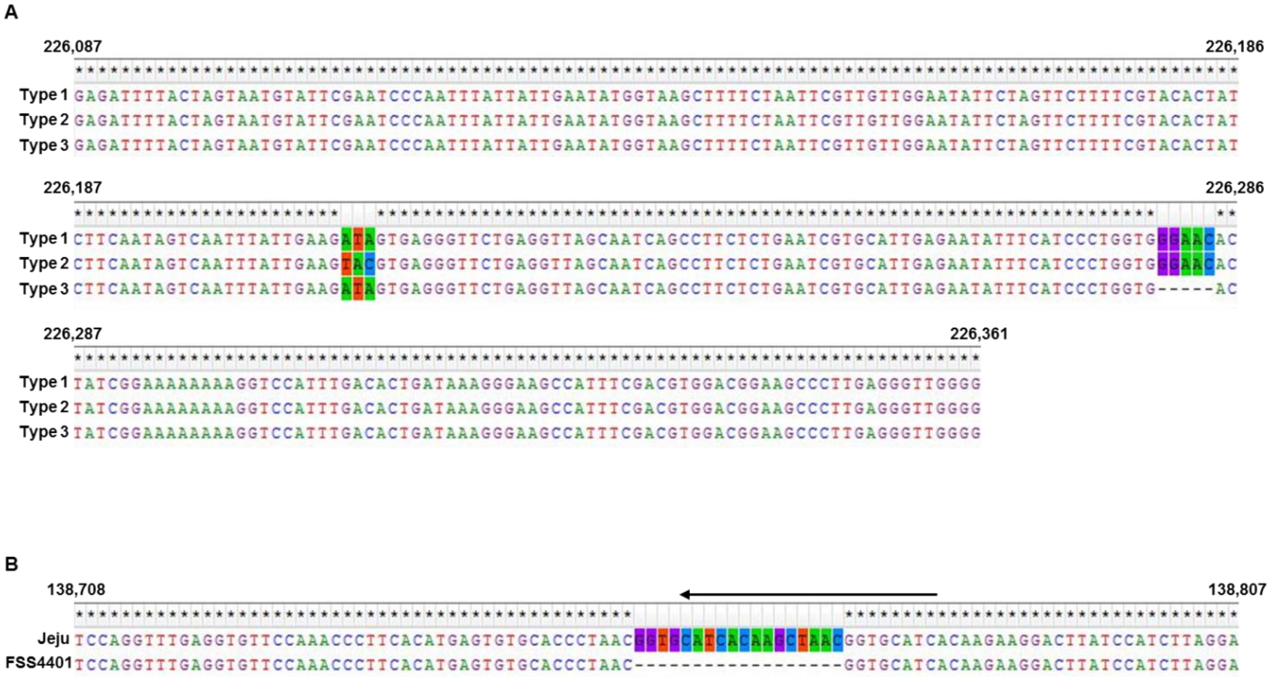

Supplementary Figure S1. SNPs and InDels used for mtDNA marker development. **(A)** Alignment of sequences for mt-227 between different genotypes of that marker. **(B)** Alignment of a portion of mt-139 between Jeju Jaerae and FS4401. The reverse primer that was designed for Jeju Jaerae-specific amplification is indicated by an arrow. The numbers above the alignments indicate the location of sequences in the Jeju Jaerae mitochondrial genome (Jo et al. 2014).

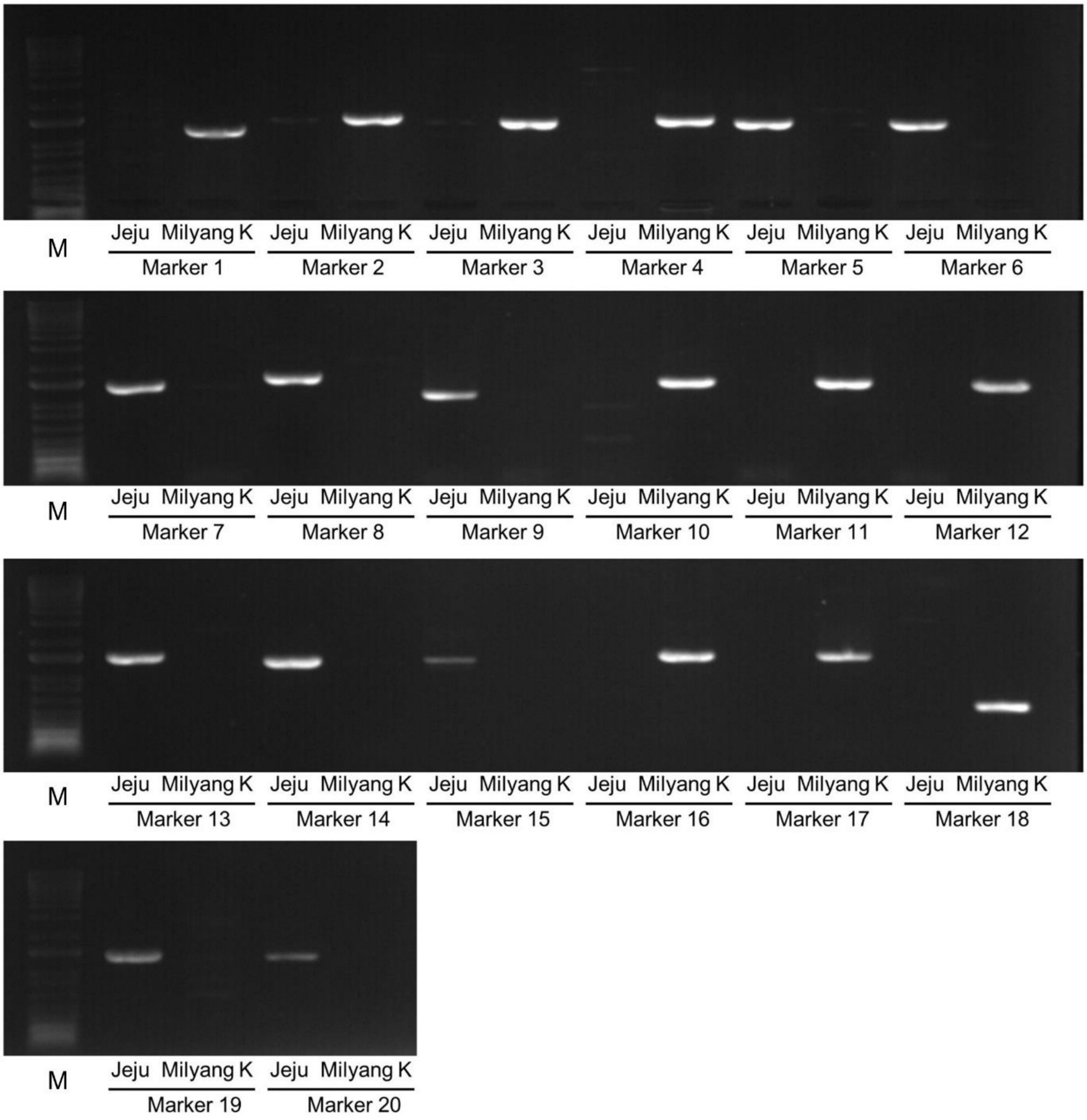

Supplementary Figure S2. Application of the 20 SCAR markers to Jeju Jaerae and Milyang K. Jeju Jaerae is abbreviated as ‘Jeju’. M refers to a size marker.

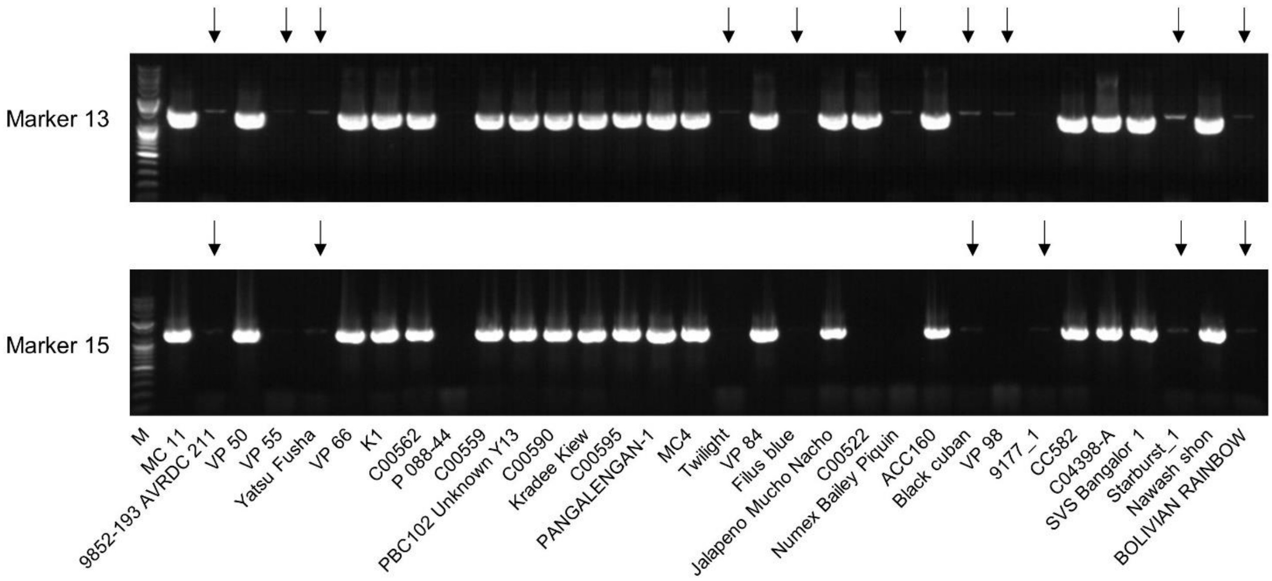

Supplementary Figure S3. Performance of SCAR markers applied to *C. annuum* accessions. Arrows indicate amplifications that were counted as “L” (low yield) in additional file 2. M refers to a size marker.

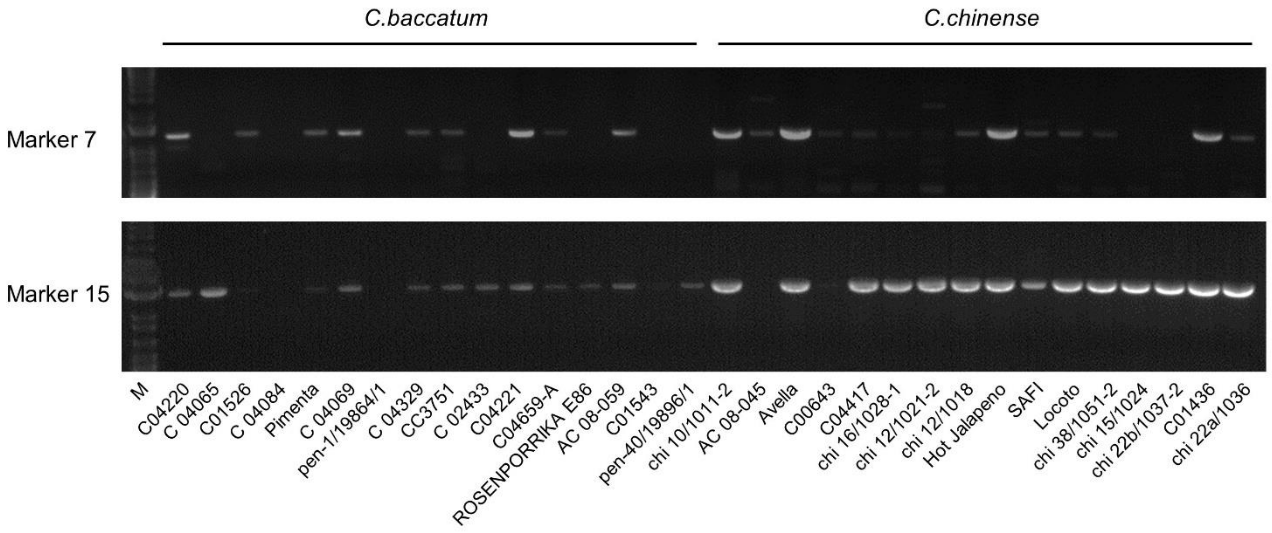

Supplementary Figure S4. Performance of SCAR markers in application to *C.baccatum* and *C.chinense* accessions. M refers to a size marker.

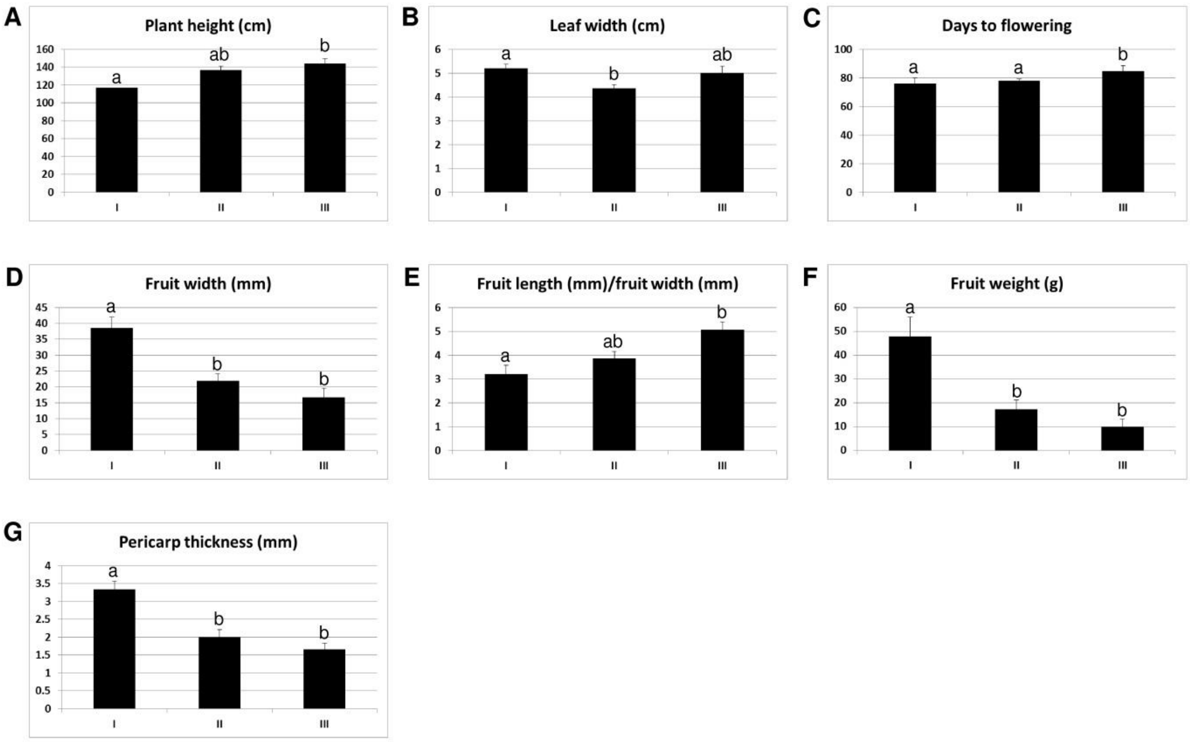

Supplementary Figure S5. Relationship of phylogenetic groups determined by application of nuclear SNP markers with phenotypes in 11 categories in 94 *C. annuum* accessions. Two accessions were excluded because they showed high levels of deviations in most phenotype categories and form a group (group IV) separated from others. This result is a subset of data reported by Lee et al. (2014). Mean values were indicated with ± SE shown as error bars. Groups regarded to be different to each other in Duncan's multiple range tests at $P < 0.05$ level were indicated by different letters above graphs.

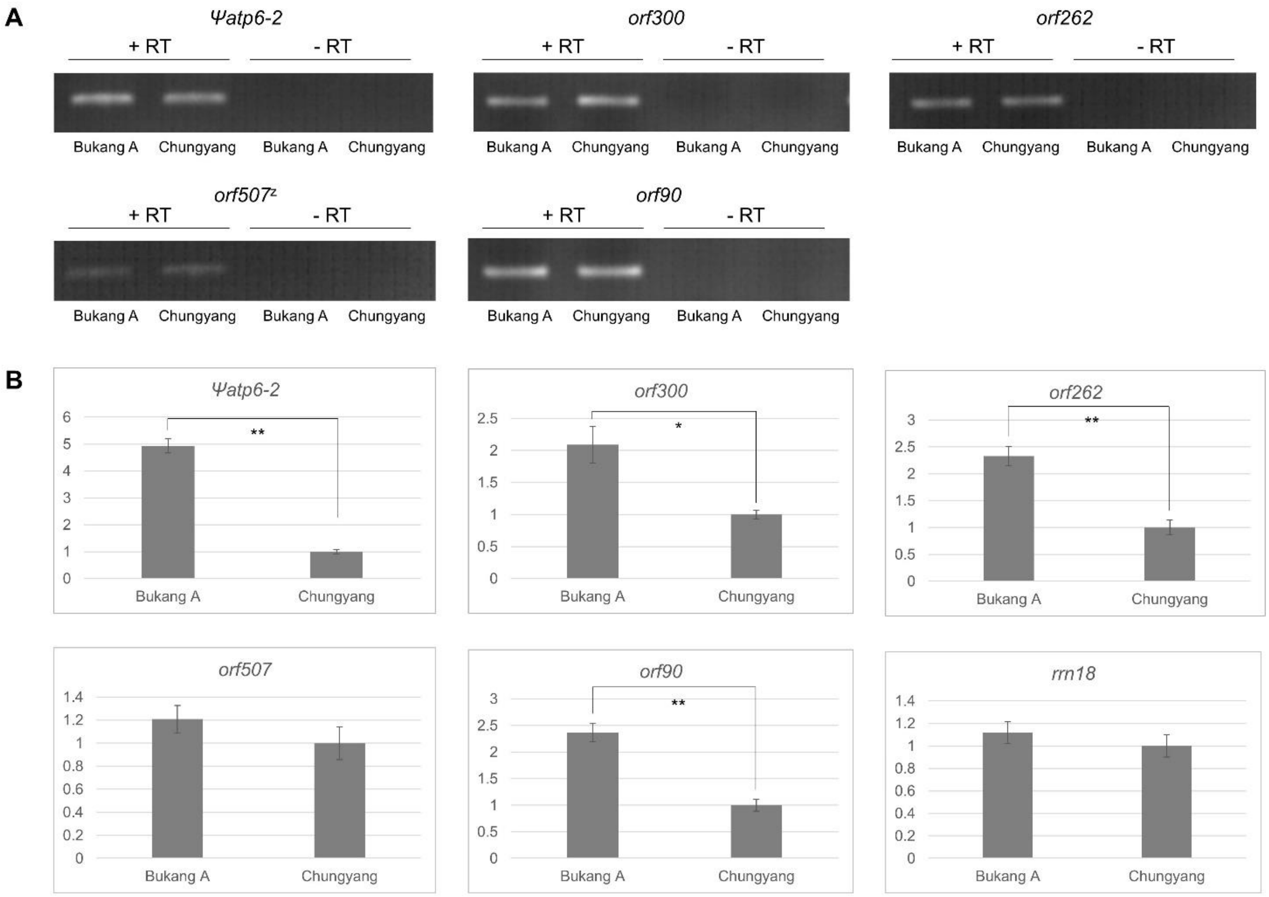

Supplementary Figure S6. Expression of *orf*s screened as candidates for the CMS-associated gene. **(A)** Result of RT-PCR analysis (30 cycle-amplification) with (+RT) or without (-RT) reverse transcriptase to confirm complete removal of mitochondrial DNA in RNA preparation. **(B)** Relative expression levels of *orf*s analyzed by qRT-PCR. Expression levels were normalized against a mitochondrial rRNA gene, *rrn26*. Expression level of *rrn18* which is another mitochondrial rRNA gene was additionally analyzed for comparison. Error bars indicate standard deviation (n=3). Statistical significance was determined by *t*-test (**p* < 0.05, ***p* < 0.01).

[z] Except for *orf507*, which was named according to its length in nucleotides in a previous study, the numbers in the names of other *orf*s represent the amino acid length of the protein products.

1.2 Supplementary Tables

Supplementary Table S1. Information about the 20 SCAR markers

Marker name	Primer sequence (5'– 3')	Amplicon length	Reference mitochondrial genome[z]	Location on reference genome
Marker 1	F: CGGAAGAGGAAGTGGCTGAAC<br>R: GTTGGCGGTGAAGTGATTCC	1,257	FS4401	173725-174981 (J)[y]<br>467849-469105[x]
Marker 2	F: GGTGGGAGGTCACAGAGAATTG<br>R: GCGCTCTTGGCTTAACAACATC	1,552	FS4401	249024-250575 (J)
Marker 3	F: CCGAATGGACGACTTGAAAAGG<br>R: CCCGAAGAGAGAAGCCCAAC	1,612	FS4401	280403-282014 (J)
Marker 4	F: GAGGTACTGCCCAAGGAAAGG<br>R: CTTTGTGGACCGACCAAGCG	1,551	FS4401	332460-334010 (J)
Marker 5	F: CACTCATTCCCATGCTAATGTAGG<br>R: GTAAGACCAAAAGCAGCCAACAC	1,463	Jeju Jaerae	23029-24491 (J)
Marker 6	F: CGGGAATGGAGAAAGGAGTAAG<br>R: GTGGAACAAAGGTCGCTCTC	1,448	Jeju Jaerae	193840-195287 (J)
Marker 7	F: CCACGAACTAAACTGCTAACTACC<br>R: GATAGGAGGTCTACGAGGTACG	1,408	Jeju Jaerae	261390-262797 (J)
Marker 8	F: GAGGTACTGCCCAAGGAAAGG<br>R: CCCCTAATTTCTTTCTCTCCTGTC	1,628	Jeju Jaerae	292199-293826 (J)
Marker 9	F: CACTGGCCTCCCATTCTTCG<br>R: CTGAATTATGGAACTTTCTCCCC	1,211	Jeju Jaerae	385308-386518 (J)
Marker 10	F: GCTCACGAATTGGATTTGAACC<br>R: GGAAGGGAAGGAAGAAGTGGAAC	1,513	FS4401	265075-266587 (S)
Marker 11	F: CCCAATGTTGACTATTGAAGATGC<br>R: GAGACACCTAATCGAAGAAGCG	1,513	FS4401	273231-274743 (S)
Marker 12	F: CGGAAGATAGGGTAGATGGTGG<br>R: CAGCCCATCCACACCTATTATG	1,512	FS4401	276735-278246 (S)
Marker 13	F: CGGCGTATGTATCCGAAAAGAG<br>R: CATGGGTGGAGTAGCCTAAGC	1,555	Jeju Jaerae	191246-192800 (S)
Marker 14	F: CTGGACTACCTCGTATCATTCC<br>R: CCAACCGGAAGAGAGTAGAATC	1,413	Jeju Jaerae	208013-209425 (S)<br>258510-259922
Marker 15	F: CGACGGTTGAGCTAGTTCAATTG<br>R: CATCGGCGATAACTATATCATCACC	1,478	Jeju Jaerae	207708-209185 (S)<br>258205-259682
Marker 16	F: GAGGAAAGGGAAGGACTAGGAAG<br>R: CAGGAAGAAGTTCACACCAGG	1,553	FS4401	304560-306112 (J)
Marker 17	F: CGCATGACTTTCCTCGGTAACC<br>R: CCATTCCGGTCGAGTAGCTC	1,547	FS4401	463229-464775 (J)
Marker 18	F: GATGTTGCAGCCCTGGAATCAC<br>R: CAACACTTCCTCGTAGGGTCAC	599	FS4401	174948-175546 (J)<br>469072-469670
Marker 19	F: CCCATGAAATGATAGCGTCCATC<br>R: GGAAAAATCACAGGGGAAGGCC	1,436	Jeju Jaerae	184024-185459 (J)
Marker 20	F: CCTGTAGCTCAGAGGATTAGAGC<br>R: CTAAATCCTCATCTTCATCTGCGG	1,366	Jeju Jaerae	241968-243333 (J)

[z] Reference genomes were reported by Jo et al. (2014).
[y] J: sequences containing rearrangement junctions
S: sequences unique to one of two lines
[x] Sequences for which locations are described two times are located within large repeated sequences.

Supplementary Table S2. Mitotypes of *C. annuum* accessions determined by markers

Accession name	Country	Group in dendro-gram (CC240)[z]	Group in dendro-gram (3,821)[y]	Mitotyping markers 1[x]	2	3	4	5	6	7	8	9	10	11	12	13	14	15	16	17	18	19	20	Classification using 20 markers	Classification using MCM1
CMV 980	HUN	I	C	H	N[w]	N	N	N	N	N	N	L	L	N	N	L	L	N	N	N	L	N	L	Group 1	Type 1
HDA295	FRA	I	C	H	N	N	N	N	N	L	N	L	N	N	N	L	L	L	N	N	L	N	N	Group 1	Type 1
Numex Mirasol	USA	I	A	H	N	N	N	N	N	N	N	L	L	N	N	L	N	N	N	N	N	N	L	Group 1	Type 1
Thai Hot	THA	II	C	H	N	N	N	N	N	N	N	N	N	N	N	N	N	N	N	N	N	N	N	Group 1	Type 1
9852-193 AVRDC 211	TWN	II	C	H	N	N	N	N	N	L	N	L	N	N	N	L	N	N	N	N	L	N	N	Group 1	Type 1
VP 55	VNM	II	D	H	N	N	N	N	N	L	N	L	L	N	L	L	N	N	N	N	L	L	N	Group 1	Type 1
Yatsu Fusha	NPL	II	C	H	N	N	N	N	N	L	N	L	N	N	N	L	N	L	N	N	L	L	N	Group 1	Type 1
Twilight	USA	IV	E	H	N	N	N	N	N	L	N	L	N	N	N	L	L	N	N	N	L	N	N	Group 1	Type 1
Filus blue	USA	II	C	H	N	N	N	N	N	N	N	L	L	N	N	L	L	N	N	N	L	N	N	Group 1	Type 1
Black cuban	USA	II	C	H	N	N	N	N	N	L	N	N	N	N	N	L	L	L	N	N	L	N	N	Group 1	Type 1
VP 98	VNM	II	D	H	N	N	N	N	N	L	N	L	N	N	N	L	N	N	N	N	L	N	N	Group 1	Type 1
9177_1	USA	II	C	H	N	N	N	N	N	N	N	N	L	N	L	N	L	N	N	N	L	N	N	Group 1	Type 1
Starburst_1	USA	I	B	H	N	N	N	N	N	L	N	N	N	N	N	L	N	L	N	N	L	N	N	Group 1	Type 1
CMV 1078/1	HUN	I	A	H	N	N	N	N	N	L	N	N	N	N	N	N	N	N	N	N	H	N	N	Group 2	Type 1
BOLIVIAN RAINBOW	BOL	II	C	H	N	N	N	N	N	L	N	N	L	N	N	L	N	L	N	N	H	L	N	Group 2	Type 1
Macskasarga	USA	I	B	H	N	N	N	N	N	N	N	L	H	H	H	N	H	H	N	N	L	N	N	Group 3	Type 1
Tura	USA	IV	E	N	H	N	N	H	H	H	H	N	L	N	L	H	H	H	N	N	L	H	H	Group 4	Type 2
Zolotoi yubileinii	MDA	I	B	N	H	N	N	H	H	H	H	N	N	N	N	H	H	H	N	N	N	H	H	Group 4	Type 2
Corne ll14	USA	I	B	N	H	N	N	H	H	H	H	N	N	N	N	H	H	H	N	N	N	H	H	Group 4	Type 2
AC 09-035	JPN	I	B	N	H	N	N	H	H	H	H	L	L	N	N	H	H	H	N	N	N	H	H	Group 4	Type 2
Canada cheese	CAN	I	B	N	H	N	N	H	H	H	H	N	N	N	N	H	H	H	N	N	N	H	H	Group 4	Type 2
Spanish Piquillo	USA	I	B	N	H	N	N	H	H	H	H	N	N	N	N	H	H	H	N	N	N	H	H	Group 4	Type 2
99-10-47	CHN	I	B	N	H	N	N	H	H	H	H	N	L	N	L	H	H	H	N	N	N	H	H	Group 4	Type 2
C01291	CRI	I	B	N	H	N	N	H	H	H	H	L	N	N	N	H	H	H	N	N	N	H	H	Group 4	Type 2
Chocolate Beauty	USA	I	B	N	H	N	N	H	H	H	H	N	N	N	N	H	H	H	N	N	N	H	H	Group 4	Type 2
Swedish	RUS	I	B	N	H	N	N	H	H	H	H	N	N	N	N	H	H	H	N	N	N	H	H	Group 4	Type 2
Malishok	RUS	I	B	N	H	N	N	H	H	H	H	L	N	N	N	H	H	H	N	N	N	H	H	Group 4	Type 2
Moldova-118	MDA	I	B	N	H	N	N	H	H	H	H	L	L	N	N	H	H	H	N	N	N	H	H	Group 4	Type 2
Volzanin	RUS	I	B	N	H	N	N	H	H	H	H	N	N	N	N	H	H	H	N	N	N	H	H	Group 4	Type 2
Zavolgski 55	RUS	I	B	N	H	N	N	H	H	H	H	L	N	N	N	H	H	H	N	N	N	H	H	Group 4	Type 2
Sweet Banana	USA	I	B	N	H	N	N	H	H	H	H	L	N	N	N	H	H	H	N	N	N	H	H	Group 4	Type 2
Uz000170	UZB	I	B	N	H	N	N	H	H	H	H	L	N	N	N	H	H	H	N	N	N	H	H	Group 4	Type 2
Dobrynya nikitich	RUS	I	B	N	H	N	N	H	H	H	H	N	N	N	N	H	H	H	N	N	N	H	H	Group 4	Type 2
UZB-HHS-2008-15	KGZ	II	A	N	H	N	N	H	H	H	H	N	L	N	N	H	H	H	N	N	N	H	H	Group 4	Type 2
Edesalma	USA	I	B	N	H	N	N	H	H	H	H	N	L	N	N	H	H	H	N	N	N	H	H	Group 4	Type 2
Kapia	BGR	II	A	N	H	N	N	H	H	H	H	N	N	N	N	H	H	H	N	N	N	H	H	Group 4	Type 2
HRF	USA	II	C	N	H	N	N	H	H	H	H	N	N	N	N	H	H	H	N	N	N	H	H	Group 4	Type 2
PI257052	COL	II	C	N	H	N	N	H	H	H	H	N	N	N	N	H	H	H	N	N	N	H	H	Group 4	Type 2
VAR.KAPIA F1-96	BGR	I	B	N	H	N	N	H	H	H	H	L	N	N	N	H	H	H	N	N	N	H	H	Group 4	Type 2
Agronomico NO.8	USA	I	B	N	H	N	N	H	H	H	H	L	L	N	N	H	H	H	N	N	N	H	N	Group 5	Type 2
ACC175	PHL	I	B	N	H	N	N	H	H	H	H	N	H	H	H	H	H	H	N	N	N	H	H	Group 6	Type 2
PI257049	COL	I	B	N	H	N	N	H	H	H	H	H	H	H	H	H	H	H	N	N	N	H	H	Group 7	Type 2
P 088-57	CHN	II	C	N	N	N	N	H	H	H	H	H	N	N	N	H	H	H	N	N	N	H	H	Group 8	Type 3
Taltos	HUN	I	B	N	N	N	N	H	H	H	H	H	L	N	N	H	H	H	N	N	N	H	H	Group 8	Type 3
WIR 1570	KAZ	II	D	N	N	N	N	H	H	H	H	H	N	N	N	H	H	H	N	N	N	H	H	Group 8	Type 3
KC 00711	USA	I	D	N	N	N	N	H	H	H	H	H	N	N	N	H	H	H	N	N	N	H	H	Group 8	Type 3
Joseon pepper	KOR	II	C	N	N	N	N	H	H	H	H	H	N	N	N	H	H	H	N	N	N	H	H	Group 8	Type 3
Namwon (Landrace)	KOR	II	C	N	N	N	N	H	H	H	H	H	N	N	N	H	H	H	N	N	N	H	H	Group 8	Type 3
KC 00016	USA	I	D	N	N	N	N	H	H	H	H	H	L	N	N	H	H	H	N	N	N	H	H	Group 8	Type 3
P 088-21	CHN	I	A	N	N	N	N	H	H	H	H	H	N	N	N	H	H	H	N	N	N	H	H	Group 8	Type 3
New Mexico	USA	I	B	N	N	N	N	H	H	H	H	H	L	N	N	H	H	H	N	N	N	H	H	Group 8	Type 3
Ikdosan	CHN	II	C	N	N	N	N	H	H	H	H	H	N	N	N	H	H	H	N	N	N	H	H	Group 8	Type 3
GWANGJU	KOR	II	C	N	N	N	N	H	H	H	H	H	L	N	N	H	H	H	N	N	N	H	H	Group 8	Type 3
gwangyang-3	KOR	II	C	N	N	N	N	H	H	H	H	H	L	N	N	H	H	H	N	N	N	H	H	Group 8	Type 3

(landrace)																									
jindo (Landrace)	KOR	II	C	N	N	N	N	H	H	H	H	H	N	N	N	H	H	H	N	N	N	H	H	Group 8	Type 3
RED & GREEN P5-4	KOR	III	D	N	N	N	N	H	H	H	H	H	N	N	N	H	H	H	N	N	N	H	H	Group 8	Type 3
hwaseong (landrace)	KOR	II	C	N	N	N	N	H	H	H	H	H	N	N	N	H	H	H	N	N	N	H	H	Group 8	Type 3
Sinheung 83-2852	KOR	II	C	N	N	N	N	H	H	H	H	H	N	L	N	H	H	H	N	N	N	H	H	Group 8	Type 3
jinju (landrace)	KOR	II	C	N	N	N	N	H	H	H	H	H	L	N	L	H	H	H	N	N	N	H	H	Group 8	Type 3
Naramurazaki	JPN	I	C	N	N	N	N	H	H	H	H	H	L	N	L	H	H	H	N	N	N	H	H	Group 8	Type 3
ulju (landrace)	KOR	II	C	N	N	N	N	H	H	H	H	H	L	N	N	H	H	H	N	N	N	H	H	Group 8	Type 3
sangju (Landrace)	KOR	II	C	N	N	N	N	H	H	H	H	H	N	N	N	H	H	H	N	N	N	H	H	Group 8	Type 3
MC11	VNM	I	C	N	N	N	N	H	H	H	H	H	N	N	N	H	H	H	N	N	N	H	H	Group 8	Type 3
VP 50	VNM	II	D	N	N	N	N	H	H	H	H	H	L	N	N	H	H	H	N	N	N	H	H	Group 8	Type 3
VP 66	VNM	II	D	N	N	N	N	H	H	H	H	H	N	N	N	H	H	H	N	N	N	H	H	Group 8	Type 3
K1	MYS	I	C	N	N	N	N	H	H	H	H	H	L	N	N	H	H	H	N	N	N	H	H	Group 8	Type 3
C00562	THA	II	C	N	N	N	N	H	H	H	H	H	L	N	N	H	H	H	N	N	N	H	H	Group 8	Type 3
C00559	THA	I	D	N	N	N	N	H	H	H	H	H	N	N	N	H	H	H	N	N	N	H	H	Group 8	Type 3
PBC102 Unknown Y13	TWN	I	C	N	N	N	N	H	H	H	H	H	L	N	L	H	H	H	L	N	N	H	H	Group 8	Type 3
C00590	THA	III	D	N	N	N	N	H	H	H	H	H	N	N	L	H	H	H	N	N	N	H	H	Group 8	Type 3
Kradee Kiew	THA	I	D	N	N	N	N	H	H	H	H	H	N	N	L	H	H	H	N	N	N	H	H	Group 8	Type 3
C00595	THA	III	D	N	N	N	N	H	H	H	H	H	N	N	N	H	H	H	N	N	N	H	H	Group 8	Type 3
PANGALENGA N-1	IDN	III	D	N	N	N	N	H	H	H	H	H	N	N	N	H	H	H	N	N	N	H	H	Group 8	Type 3
MC4	MYS	III	C	N	N	N	N	H	H	H	H	H	N	N	N	H	H	H	N	N	N	H	H	Group 8	Type 3
VP 84	VNM	II	D	N	N	N	N	H	H	H	H	H	L	N	N	H	H	H	N	N	N	H	H	Group 8	Type 3
Jalapeno Mucho Nacho	USA	I	D	N	N	N	N	H	H	H	H	H	N	N	N	H	H	H	N	N	N	H	H	Group 8	Type 3
C00522	PHL	III	D	N	N	N	N	H	H	H	H	H	N	N	N	H	H	H	N	N	N	H	H	Group 8	Type 3
ACC160	PHL	I	C	N	N	N	N	H	H	H	H	H	L	N	L	H	H	H	N	N	N	H	H	Group 8	Type 3
CC582	NPL	-	-	N	N	N	N	H	H	H	H	H	N	N	N	H	H	H	N	N	N	H	H	Group 8	Type 3
SVS Bangalor 1	IND	II	C	N	N	N	N	H	H	H	H	H	L	N	L	H	H	H	N	N	N	H	H	Group 8	Type 3
Nawash shon	PAK	II	D	N	N	N	N	H	H	H	H	H	L	N	N	H	H	H	N	N	N	H	H	Group 8	Type 3
5502	USA	I	B	N	N	N	N	H	H	H	N	N	N	N	N	H	H	H	N	N	N	H	H	Group 9	Type 3
Greygo	HUN	I	B	N	N	N	N	H	H	H	N	N	N	N	N	H	H	H	N	N	N	H	H	Group 9	Type 3
Hai li 10	CHN	I	B	N	N	N	N	H	H	H	H	N	N	N	N	H	H	H	N	N	N	H	H	Group 10	Type 3
09G111	USA	I	B	N	N	N	N	H	H	H	H	N	N	N	N	H	H	H	N	N	N	H	H	Group 10	Type 3
Chorbadjiyska	BGR	I	B	N	N	N	N	H	H	H	H	H	N	N	N	H	H	H	N	N	N	H	L	Group 11	Type 3
Gyeonggiyangpyeong-1985-gochu103410	KOR	II	C	N	N	N	N	H	H	H	H	H	N	N	N	H	H	H	N	N	N	H	N	Group 11	Type 3
Guajillo ancho	USA	I	B	H	H	H	H	N	N	N	N	N	H	H	H	N	N	N	H	H	H	N	N	Group 12	Type 4
KC 00003	USA	II	D	H	H	H	H	N	N	L	N	L	H	H	H	N	N	N	H	H	H	N	N	Group 12	Type 4
P 97633	KOR	III	C	H	H	H	H	N	N	N	N	N	H	H	H	N	N	N	H	H	H	N	N	Group 12	Type 4
P 088-44	CHN	II	C	H	H	H	H	N	N	N	N	L	H	H	H	N	N	N	H	H	H	N	N	Group 12	Type 4
Yellow mushroom	USA	I	A	N	N	N	N	N	N	H	N	L	N	N	N	N	H	H	L	N	N	L	N	Group 13	Type 5
UZB-GJG-1999-51	UZB	II	D	N	N	N	N	N	N	H	N	L	N	N	N	L	H	H	L	N	N	L	L	Group 13	Type 5
C04398-A	ARG	II	C	N	N	N	N	N	N	H	N	N	L	N	L	N	H	H	L	N	N	N	N	Group 13	Type 5
Mulato	MEX	I	B	N	N	N	N	H	L	H	H	N	L	N	N	H	H	H	N	N	N	H	H	Group 14	Type 5
Numex Bailey Piquin	USA	II	D	L	N	N	N	N	N	N	N	N	N	N	N	N	L	N	N	N	N	N	N	Group 15	Type 5

z Classification based on phylogenetic analysis of 240 accessions from a core collection based on 48 nuclear SNP markers [Lee et al. (2016)]

y Classification based on population structure analysis of 3,821 accessions based on 48 nuclear SNP markers [Lee et al. (2016)]

x Markers with underlines were designed from FS4401; others were designed from Jeju Jaerae.

w H: amplifications with high yield
L: amplifications with low yield
N: no amplification

Supplementary Table S3. Application of mtDNA markers to *C. chinense*, *C. frutescens*, and *C. baccatum* accessions

Species	Accession name	Country	Group in dendro-gram (CC240)[z]	Group in dendro-gram (3,821)[y]	Mitotyping markers 1[x]	2	3	4	5	6	7	8	9	10	11	12	13	14	15	16	17	18	19	20
C. chinense	Locoto	BOL	V	E	U[w]	N	N	N	N	U	U	N	U	S	N	U	U	S	S	N	N	N	N	N
C. chinense	chi 12/1018	HUN	V	E	U	N	N	N	N	N	U	N	U	S	U	S	U	S	S	N	N	N	N	N
C. chinense	chi 22a/1036	HUN	V	E	U	U	N	N	N	U	U	N	N	S	U	S	U	S	S	N	N	N	N	N
C. chinense	C00643	HND	IV	E	N	U	N	N	N	N	U	N	U	S	S	S	N	N	N	N	N	U	N	N
C. chinense	chi 16/1028-1	HUN	V	E	N	N	N	N	N	N	U	N	U	S	S	S	N	N	S	N	N	U	N	N
C. chinense	chi 12/1021-2	HUN	V	E	U	N	N	N	N	U	U	N	N	S	S	S	U	S	S	N	N	N	N	N
C. chinense	chi 38/1051-2	HUN	V	E	U	N	N	N	N	U	U	N	U	S	S	S	N	S	S	N	N	N	N	N
C. chinense	chi 15/1024	HUN	V	E	N	U	N	N	N	U	N	N	N	S	S	S	N	S	S	N	N	N	N	N
C. chinense	chi 22b/1037-2	HUN	V	E	U	N	N	N	N	U	N	N	N	S	S	S	N	S	S	N	N	N	N	N
C. chinense	chi 10/1011-2	HUN	V	E	U	N	N	N	N	U	S	N	U	S	S	S	U	S	S	N	N	N	S	N
C. chinense	Hot Jalapeno	MEX	IV	E	U	N	N	N	S	S	S	N	S	U	U	U	S	S	S	N	N	N	S	S
C. chinense	C01436	MEX	IV	E	U	N	N	N	S	S	S	N	S	U	U	U	S	S	S	N	N	U	S	S
C. chinense	SAFI	SEN	V	E	S	N	N	N	N	N	U	N	U	U	S	S	U	S	U	N	N	U	N	N
C. chinense	AC 08-045	PER	IV	E	S	N	N	N	N	N	U	N	U	S	S	S	U	N	N	N	N	N	U	N
C. chinense	C04417	BOL	V	E	S	N	N	N	N	U	U	N	U	S	S	S	U	S	S	N	N	N	N	N
C. chinense	Avella	USA	IV	E	S	N	N	N	S	S	S	N	S	S	U	N	S	S	S	N	N	N	S	S
C. frutescens	PI 555644	GTM	-	-	N	N	N	N	N	N	N	N	N	S	N	N	N	S	S	N	N	U	N	N
C. frutescens	C 04750-B	ECU	-	-	U	N	N	N	N	N	N	N	N	S	N	N	N	S	S	N	N	N	U	N
C. frutescens	C 04612	CRI	-	-	U	U	N	N	N	N	N	N	N	S	N	N	N	S	S	N	N	U	U	N
C. frutescens	Lombok	IDN	-	-	N	N	N	N	N	N	U	N	N	S	N	N	U	S	S	N	N	U	U	N
C. frutescens	C 04851	CRI	-	-	N	N	N	N	N	N	N	N	N	S	N	N	N	S	S	N	N	U	N	N
C. frutescens	KC01288	LAO	V	E	N	N	N	N	N	N	U	N	N	S	N	N	U	S	S	N	N	U	N	N
C. frutescens	C00683	GTM	IV	E	N	U	N	N	N	N	N	N	N	S	N	S	N	S	N	N	N	U	U	N
C. frutescens	KC01293	LAO	V	E	N	U	N	N	N	N	N	N	N	S	N	S	N	S	S	N	N	U	N	N
C. frutescens	AC 08-010	CRI	IV	E	U	N	N	N	N	N	U	N	U	S	N	S	U	S	S	N	N	U	U	N
C. frutescens	GREEN LEAF VARIETY	USA	V	E	U	N	N	N	N	N	N	N	N	S	N	S	N	S	S	N	N	U	S	N
C. frutescens	KC01303	LAO	IV	E	U	N	N	N	N	N	U	N	U	S	S	S	U	S	S	N	N	U	N	N
C. frutescens	P 82005	LAO	IV	E	U	N	N	N	N	N	S	N	N	S	N	N	N	S	N	N	N	U	U	N
C. frutescens	EKIRIKE	NGA	IV	E	N	U	N	N	N	N	S	N	N	S	N	N	N	S	S	N	N	N	N	N
C. frutescens	NUM-6 L.G.G.-J.L.	CRI	-	-	U	U	N	N	S	N	S	N	N	S	N	N	S	S	S	N	N	U	S	U
C. frutescens	VTP 220	LAO	V	E	S	U	N	N	N	U	S	N	N	S	N	S	S	S	N	N	N	U	U	N
C. frutescens	Toko Subur	IDN	V	E	S	N	N	N	N	U	S	N	U	S	S	S	U	S	S	S	N	S	U	N
C. baccatum	C 04084	MEX			N	U	N	N	N	N	N	N	N	N	N	N	N	N	N	N	N	N	N	N
C. baccatum	pen-1/19864/1	HUN	V	E	N	N	N	N	N	N	N	N	N	N	N	N	U	N	N	U	N	N	N	N
C. baccatum	pen-40/19896/1	HUN	V	E	N	U	N	N	N	N	N	N	N	U	U	U	U	U	U	U	N	N	N	N
C. baccatum	C 02433	PER	-	-	N	U	N	N	N	N	N	N	U	S	U	N	N	U	U	U	N	N	N	N
C. baccatum	C 04065	ARG	-	-	N	N	N	N	N	N	N	N	N	S	S	S	U	S	U	U	N	N	U	N
C. baccatum	C01543	SLV	IV	E	N	U	N	N	U	N	N	N	S	N	N	N	S	S	N	U	N	N	N	N
C. baccatum	C04220	PHL	IV	E	N	U	N	N	N	N	S	N	U	U	U	U	U	U	U	U	N	N	N	N
C. baccatum	C01526	USA	IV	E	N	N	N	N	N	N	S	N	N	U	U	U	N	U	U	N	N	N	N	N
C. baccatum	Pimenta	BRA	IV	E	N	N	N	N	N	N	S	N	U	N	N	N	U	U	U	N	N	N	U	N
C. baccatum	C 04329	BRA	-	-	N	N	N	N	N	N	S	N	U	U	U	U	U	U	U	U	N	N	N	N
C. baccatum	C04659-A	SUR	V	E	U	N	N	N	N	N	S	N	U	U	U	U	U	U	U	U	N	N	N	N
C. baccatum	ROSENPORRIKA E86	AUT	IV	E	S	U	N	N	N	N	N	N	U	U	N	U	U	U	U	U	N	N	N	N
C. baccatum	C 04069	BOL	-	-	S	N	N	N	N	N	S	N	U	U	U	N	U	U	U	U	N	N	N	N
C. baccatum	CC3751	ARG	-	-	S	U	N	N	N	N	S	N	U	N	N	U	U	U	U	U	N	N	N	N
C. baccatum	AC 08-059	NLD	IV	E	S	N	N	N	N	N	S	N	U	N	N	N	U	U	U	U	N	N	N	N
C. baccatum	C04221	GUY	IV	E	S	U	N	N	N	N	S	N	U	U	U	U	S	U	U	U	N	N	U	N

[z] Classification based on phylogenetic analysis of 240 core-collection accessions using 48 nuclear SNP markers [Lee et al. (2016)]
[y] Classification based on population structure analysis of 3,821 accessions using 48 nuclear SNP markers [Lee et al. (2016)]
[x] Markers with underlines were designed from FS4401 while others were from Jeju Jaerae.
[w] S: amplifications with high yield
U: amplifications with intermediate or low yield
N: no amplification

Supplementary Table S4. Information about MCM1, a multiplex marker for mitotyping

Primer combination name	Primer sequence (5' to 3')	Amplicon length	Original marker	Reference mitochondrial genome[z]
Marker 1	F: CGGAAGAGGAAGTGGCTGAAC<br>R: GTTGGCGGTGAAGTGATTCC	1,257	Marker 1	FS4401
Marker 2	F: GGTGGGAGGTCACAGAGAATTG<br>R: GCGCTCTTGGCTTAACAACATC	1,552	Marker 2	FS4401
Marker 3-S	F: CCGAATGGACGACTTGAAAAGG<br>R: GAACCTGCCATAGTGAAGCGC	963	Marker 3	FS4401
Marker 6-S	F: GGGATTCGTTTTGGGCATCCTC<br>R: CACTGTTGTTGGGAGAGTAGTTCC	520	Marker 6	Jeju

[z] Reference genomes were reported by Jo et al. (2014).

Supplementary Table S5. Information about markers based on sequence polymorphisms in mitochondrial and chloroplast DNA

Marker name	Primer sequence (5’ to 3’ direction)	Tm (°C)	Origin of sequence
mt-227	F: CAACATCCAATGGGTCATTCAC<br>R: GAAGGAGGGGACCTTTTCTC	60	mtDNA
mt-217	F: GAAGATAGAGACAGAGAAGGTTAGG<br>R: CTACTAGACGAAGGCGAAGC	62	mtDNA
PepRpl	F: AATCCGTTATTTGAATGCATT T<br>R: GTTAAACCGGGGCGAATAC	62	cpDNA

Supplementary Table S6. Information about FS4401-specific *orfs* in comparison with Jeju Jaerae. Only *orf*s encoding putative proteins ≥ 50 amino acids in length and predicted to have a transmembrane domain are listed.

orf name	Location on FS4401 mtDNA	RPKM in 121A	RPKM in 121C	Sequences of primers (5'– 3') for qRT-PCR analysis	Sequences of primers (5'– 3') for DNA amplification analysis
Ψatp6-2	262743-263687	76.93	7.22	F:CCAGTTCCAGTAATATAGCTTCGC<br>R:GGTCCAGACCTTCAAAAAAACC	F:TGGATCTCGCTATTAACCAC<br>R:GTAGTTCATTCGGACCTAGTAG
orf300	303065-302163	3.39	0.54	F:GTTGAACTTGAGCAGAACGAGATG<br>R:CCTGAAAGAATGTCTCTCGAACG	F:GACTGAATCGTGTGCGAGAATTATC<br>R:CCGAAAAAAAGAAGCGACTACCC
orf262	270994-271782	6.02	0.56	F:CTCACAGGCGAGGAGTTCAC<br>R:GACGCAGACGAGACGGATTG	F:GGGATAAAAGGCTGGTCCCAAC<br>R:CAGGTCTTGTTTCTGATGGACTTC
orf507	249932-250438	103.13	76.33	F:CAGATGGAACGAGTATGCACATC<br>R:CTGCAACGGCGACAGACGAA	F:GGTGGGAGGTCACAGAGAATTG<br>R:GCGCTCTTGGCTTAACAACATC
orf108a	23903-23577	0	0.13	-	-
orf90	280320-280048	6.05	0.97	F:GTCTTTCGGTCTTGATCTGAGC<br>R:CTTAGACCTTTAGACCTGACCAAG	F:CAGTTAATCAGGGCTTAGGCCC<br>R:GATTCGATCATTCAAGTCGGATAGC
orf78	334324-334088	0.17	0		-
orf77	232814-232581	0.69	0		-
orf76	264637-264407	0.17	0		-
orf70	277584-277372	0.57	0		-
orf68	267159-266953	0.39	0		-
orf60	334337-334155	0	0		-
orf57	23601-23428	0.23	0		-
orf54	256432-256596	0	0		-
orf51	274674-274519	0.52	0		-
orf50	251006-250854	0.53	0		-

Supplementary Table S7. Polymorphism information content (PIC) values of twenty SCAR markers and MCM1

Marker name	Number of haplotypes	PIC
Marker 1	2	0.33
Marker 2	2	0.43
Marker 3	2	0.08
Marker 4	2	0.08
Marker 5	2	0.38
Marker 6	2	0.39
Marker 7	2	0.34
Marker 8	2	0.39
Marker 9	2	0.49
Marker 10	2	0.14
Marker 11	2	0.14
Marker 12	2	0.14
Marker 13	2	0.38
Marker 14	2	0.33
Marker 15	2	0.33
Marker 16	2	0.08
Marker 17	2	0.08
Marker 18	2	0.12
Marker 19	2	0.38
Marker 20	2	0.40
MCM1	5	0.67

Supplementary Table S8. Application of markers based on sequence polymorphism in mtDNA and cpDNA to *Capsicum* germplasms

Accession name	Species	Classification using 20 markers	Classification using MCM1	Genotyping using markers mt-227	mt-139	PepRpl
Numex Mirasol	*C.annuum*	Group 1	Type 1	Type 1	N	N
Thai Hot	*C.annuum*	Group 1	Type 1	Type 1	N	N
Twilight	*C.annuum*	Group 1	Type 1	Type 1	N	N
Starburst_1	*C.annuum*	Group 1	Type 1	Type 1	N	N
Yatsu Fusha	*C.annuum*	Group 1	Type 1	Type 1	N	N
CMV 1078/1	*C.annuum*	Group 2	Type 1	Type 1	N	N
BOLIVIAN RAINBOW	*C.annuum*	Group 2	Type 1	Type 1	N	N
Macskasarga	*C.annuum*	Group 3	Type 1	Type 1	N	N
Tura	*C.annuum*	Group 4	Type 2	Type 2	Y	Y
AC 09-035	*C.annuum*	Group 4	Type 2	Type 2	Y	Y
Volzanin	*C.annuum*	Group 4	Type 2	Type 2	Y	Y
Kapia	*C.annuum*	Group 4	Type 2	Type 2	Y	Y
PI257052	*C.annuum*	Group 4	Type 2	Type 2	Y	Y
Agronomico NO.8	*C.annuum*	Group 5	Type 2	Type 2	Y	Y
ACC175	*C.annuum*	Group 6	Type 2	Type 2	Y	Y
PI257049	*C.annuum*	Group 7	Type 2	Type 2	Y	Y
Taltos	*C.annuum*	Group 8	Type 3	Type 2	Y	Y
Naramurazaki	*C.annuum*	Group 8	Type 3	Type 2	Y	N
hwaseong (landrace)	*C.annuum*	Group 8	Type 3	Type 2	Y	N
Jalapeno Mucho Nacho	*C.annuum*	Group 8	Type 3	Type 2	Y	Y
SVS Bangalor 1	*C.annuum*	Group 8	Type 3	Type 2	Y	Y
5502	*C.annuum*	Group 9	Type 3	Type 2	Y	Y
Greygo	*C.annuum*	Group 9	Type 3	Type 2	Y	Y
Hai li 10	*C.annuum*	Group 10	Type 3	Type 2	Y	Y
09G111	*C.annuum*	Group 10	Type 3	Type 2	Y	N
Chorbadjiyska	*C.annuum*	Group 11	Type 3	Type 2	Y	Y
Gyeonggiyangpyeong-1985-gochu103410	*C.annuum*	Group 11	Type 3	Type 2	Y	N
Guajillo ancho	*C.annuum*	Group 12	Type 4	Type 1	N	Y
KC 00003	*C.annuum*	Group 12	Type 4	Type 1	N	Y
P 97633	*C.annuum*	Group 12	Type 4	Type 1	N	Y
P 088-44	*C.annuum*	Group 12	Type 4	Type 1	N	Y
Yellow mushroom	*C.annuum*	Group 13	Type 5	Type 1	N	N
UZB-GJG-1999-51	*C.annuum*	Group 13	Type 5	Type 1	N	Y
C04398-A	*C.annuum*	Group 13	Type 5	Type 1	N	Y
Mulato	*C.annuum*	Group 14	Type 5	Type 2	Y	N
Numex Bailey Piquin	*C.annuum*	Group 15	Type 5	Type 1	N	N
BukangA	*C.annuum* (CMS)	Group 12	Type 4	Type 1	N	Y
Chungyang A	*C.annuum* (CMS)	Group 12	Type 4	Type 1	N	Y
KA-2	*C.annuum* (CMS)	Group 12	Type 4	Type 1	N	Y
Manitta	*C.annuum* (CMS)	Group 12	Type 4	Type 1	N	Y
P 82005	*C. frutescens*	-[z]	-	Type 3	N	N
NUM-6 L.G.G.-J.L.	*C. frutescens*	-	-	Type 3	N	N
C00683	*C. frutescens*	-	-	Type 3	N	N
PI 555644	*C. frutescens*	-	-	Type 3	N	N
GREEN LEAF VARIETY	*C. frutescens*	-	-	Type 3	N	N
C 04750-B	*C. frutescens*	-	-	Type 3	N	N
VTP 220	*C. frutescens*	-	-	Type 3	N	N
C 04612	*C. frutescens*	-	-	Type 3	N	N
KC01293	*C. frutescens*	-	-	Type 3	N	N
Lombok	*C. frutescens*	-	-	Type 3	N	N
AC 08-010	*C. frutescens*	-	-	Type 3	N	N
C 04851	*C. frutescens*	-	-	Type 3	N	N
KC01303	*C. frutescens*	-	-	Type 3	N	N
KC01288	*C. frutescens*	-	-	Type 3	N	N
Toko Subur	*C. frutescens*	-	-	Type 3	N	N
EKIRIKE	*C. frutescens*	-	-	Type 3	N	N
chi 10/1011-2	*C. chinense*	-	-	Type 3	N	N
AC 08-045	*C. chinense*	-	-	Type 3	N	N
Avella	*C. chinense*	-	-	Type 2	Y	Y
C00643	*C. chinense*	-	-	Type 3	N	N
C04417	*C. chinense*	-	-	Type 3	N	N
chi 16/1028-1	*C. chinense*	-	-	Type 3	N	N

chi 12/1021-2	*C. chinense*	-	-	Type 3	N	N
chi 12/1018	*C. chinense*	-	-	Type 3	N	N
Hot Jalapeno	*C. chinense*	-	-	Type 2	Y	Y
SAFI	*C. chinense*	-	-	Type 1	N	N
Locoto	*C. chinense*	-	-	Type 1	N	N
chi 38/1051-2	*C. chinense*	-	-	Type 3	N	N
chi 15/1024	*C. chinense*	-	-	Type 3	N	N
chi 22b/1037-2	*C. chinense*	-	-	Type 3	N	N
C01436	*C. chinense*	-	-	Type 2	N	Y
chi 22a/1036	*C. chinense*	-	-	Type 3	N	N
C04220	*C. baccatum*	-	-	ND[y]	N	N
C 04065	*C. baccatum*	-	-	ND	N	N
C01526	*C. baccatum*	-	-	ND	N	N
C 04084	*C. baccatum*	-	-	ND	N	N
Pimenta	*C. baccatum*	-	-	ND	N	N
C 04069	*C. baccatum*	-	-	ND	N	N
pen-1/19864/1	*C. baccatum*	-	-	ND	N	N
C 04329	*C. baccatum*	-	-	ND	N	N
CC3751	*C. baccatum*	-	-	ND	N	N
C 02433	*C. baccatum*	-	-	ND	N	N
C04221	*C. baccatum*	-	-	ND	N	N
C04659-A	*C. baccatum*	-	-	ND	N	N
ROSENPORRIKA E86	*C. baccatum*	-	-	ND	N	N
AC 08-059	*C. baccatum*	-	-	ND	N	N
C01543	*C. baccatum*	-	-	ND	N	N
pen-40/19896/1	*C. baccatum*	-	-	ND	N	N

Supplementary Table S9. Amplification *orf*s that are candidates for CMS-associated genes

Accession name	Species	Classification using 20 markers	Classification using MCM1	Genotyping using markers				
				Ψatp6-2	*orf300*	*orf262*	*orf507*	*orf90*
CMV 980	*C.annuum*	Group 1	Type 1	N	Y	N	N	N
HDA295	*C.annuum*	Group 1	Type 1	N	Y	N	N	N
Numex Mirasol	*C.annuum*	Group 1	Type 1	N	Y	N	N	N
Thai Hot	*C.annuum*	Group 1	Type 1	N	Y	N	N	N
9852-193 AVRDC 211	*C.annuum*	Group 1	Type 1	N	Y	N	N	N
VP 55	*C.annuum*	Group 1	Type 1	N	Y	N	N	N
Yatsu Fusha	*C.annuum*	Group 1	Type 1	N	Y	N	N	N
Twilight	*C.annuum*	Group 1	Type 1	N	Y	N	N	N
Filus blue	*C.annuum*	Group 1	Type 1	N	Y	N	N	N
Black cuban	*C.annuum*	Group 1	Type 1	N	Y	N	N	N
VP 98	*C.annuum*	Group 1	Type 1	N	Y	N	N	N
9177_1	*C.annuum*	Group 1	Type 1	N	Y	N	N	N
Starburst_1	*C.annuum*	Group 1	Type 1	N	Y	N	N	N
CMV 1078/1	*C.annuum*	Group 2	Type 1	N	Y	N	N	N
BOLIVIAN RAINBOW	*C.annuum*	Group 2	Type 1	N	Y	N	N	N
Macskasarga	*C.annuum*	Group 3	Type 1	N	Y	N	N	N
Tura	*C.annuum*	Group 4	Type 2	N	N	N	Y	N
Zolotoi yubileinii	*C.annuum*	Group 4	Type 2	N	N	N	Y	N
Corne ll14	*C.annuum*	Group 4	Type 2	N	Y	N	Y	N
AC 09-035	*C.annuum*	Group 4	Type 2	N	Y	N	Y	N
Canada cheese	*C.annuum*	Group 4	Type 2	N	N	N	Y	N
Spanish Piquillo	*C.annuum*	Group 4	Type 2	N	N	N	Y	N
99-10-47	*C.annuum*	Group 4	Type 2	N	N	N	Y	N
C01291	*C.annuum*	Group 4	Type 2	N	Y	N	Y	N
Chocolate Beauty	*C.annuum*	Group 4	Type 2	N	N	N	Y	N
Swedish	*C.annuum*	Group 4	Type 2	N	N	N	Y	N
Malishok	*C.annuum*	Group 4	Type 2	N	N	N	Y	N
Moldova-118	*C.annuum*	Group 4	Type 2	N	Y	N	Y	N
Volzanin	*C.annuum*	Group 4	Type 2	N	N	N	Y	N
Zavolgski 55	*C.annuum*	Group 4	Type 2	N	N	N	Y	N
Sweet Banana	*C.annuum*	Group 4	Type 2	N	N	N	Y	N
Uz000170	*C.annuum*	Group 4	Type 2	N	N	N	Y	N
Dobrynya nikitich	*C.annuum*	Group 4	Type 2	N	Y	N	Y	N
UZB-HHS-2008-15	*C.annuum*	Group 4	Type 2	N	W	N	Y	N
Edesalma	*C.annuum*	Group 4	Type 2	N	Y	N	Y	N
Kapia	*C.annuum*	Group 4	Type 2	N	N	N	Y	N
HRF	*C.annuum*	Group 4	Type 2	N	Y	N	Y	N
PI257052	*C.annuum*	Group 4	Type 2	N	N	N	Y	N
VAR.KAPIA F1-96	*C.annuum*	Group 4	Type 2	N	Y	N	Y	N
Agronomico NO.8	*C.annuum*	Group 5	Type 2	N	N	N	Y	N
ACC175	*C.annuum*	Group 6	Type 2	N	N	N	Y	N
PI257049	*C.annuum*	Group 7	Type 2	N	N	N	Y	N
P 088-57	*C.annuum*	Group 8	Type 3	N	N	N	N	N
Taltos	*C.annuum*	Group 8	Type 3	N	N	N	N	N
WIR 1570	*C.annuum*	Group 8	Type 3	N	N	N	N	N
KC 00711	*C.annuum*	Group 8	Type 3	N	Y	N	N	N
Joseon pepper	*C.annuum*	Group 8	Type 3	N	Y	N	N	N
Namwon (Landrace)	*C.annuum*	Group 8	Type 3	N	N	N	N	N
KC 00016	*C.annuum*	Group 8	Type 3	N	N	N	N	N
P 088-21	*C.annuum*	Group 8	Type 3	N	N	N	N	N
New Mexico	*C.annuum*	Group 8	Type 3	N	N	N	N	N
Ikdosan	*C.annuum*	Group 8	Type 3	N	N	N	N	N
GWANGJU	*C.annuum*	Group 8	Type 3	N	N	N	N	N
gwangyang-3 (landrace)	*C.annuum*	Group 8	Type 3	N	Y	N	N	N
jindo (Landrace)	*C.annuum*	Group 8	Type 3	N	N	N	N	N
RED & GREEN P5-4	*C.annuum*	Group 8	Type 3	N	Y	N	N	N
hwaseong (landrace)	*C.annuum*	Group 8	Type 3	N	N	N	N	N
Sinheung 83-2852	*C.annuum*	Group 8	Type 3	N	Y	N	N	N
jinju (landrace)	*C.annuum*	Group 8	Type 3	N	Y	N	N	N
Naramurazaki	*C.annuum*	Group 8	Type 3	N	N	N	N	N
ulju (landrace)	*C.annuum*	Group 8	Type 3	N	Y	N	N	N
sangju (Landrace)	*C.annuum*	Group 8	Type 3	N	Y	N	N	N
MC11	*C.annuum*	Group 8	Type 3	N	N	N	N	N
VP 50	*C.annuum*	Group 8	Type 3	N	N	N	N	N
VP 66	*C.annuum*	Group 8	Type 3	N	N	N	N	N

K1	*C.annuum*	Group 8	Type 3	N	N	N	N	N
C00562	*C.annuum*	Group 8	Type 3	N	N	N	N	N
C00559	*C.annuum*	Group 8	Type 3	N	N	N	N	N
PBC102 Unknown Y13	*C.annuum*	Group 8	Type 3	N	W	N	N	N
C00590	*C.annuum*	Group 8	Type 3	N	N	N	N	N
Kradee Kiew	*C.annuum*	Group 8	Type 3	N	N	N	N	N
C00595	*C.annuum*	Group 8	Type 3	N	N	N	N	N
PANGALENGAN-1	*C.annuum*	Group 8	Type 3	N	N	N	N	N
MC4	*C.annuum*	Group 8	Type 3	N	N	N	N	N
VP 84	*C.annuum*	Group 8	Type 3	N	N	N	N	N
Jalapeno Mucho Nacho	*C.annuum*	Group 8	Type 3	N	N	N	N	N
C00522	*C.annuum*	Group 8	Type 3	N	N	N	N	N
ACC160	*C.annuum*	Group 8	Type 3	N	N	N	N	N
CC582	*C.annuum*	Group 8	Type 3	N	Y	N	N	N
SVS Bangalor 1	*C.annuum*	Group 8	Type 3	N	W	N	N	N
Nawash shon	*C.annuum*	Group 8	Type 3	N	N	N	N	N
5502	*C.annuum*	Group 9	Type 3	N	Y	N	N	N
Greygo	*C.annuum*	Group 9	Type 3	N	N	N	N	N
Hai li 10	*C.annuum*	Group 10	Type 3	N	N	N	N	N
09G111	*C.annuum*	Group 10	Type 3	N	N	N	N	N
Chorbadjiyska	*C.annuum*	Group 11	Type 3	N	N	N	N	N
Gyeonggiyangpyeong-1985-gochu103410	*C.annuum*	Group 11	Type 3	N	Y	N	N	N
Guajillo ancho	*C.annuum*	Group 12	Type 4	Y	Y	Y	Y	Y
KC 00003	*C.annuum*	Group 12	Type 4	Y	Y	Y	Y	Y
P 97633	*C.annuum*	Group 12	Type 4	Y	Y	Y	Y	Y
P 088-44	*C.annuum*	Group 12	Type 4	Y	Y	Y	Y	Y
Yellow mushroom	*C.annuum*	Group 13	Type 5	N	N	N	N	N
UZB-GJG-1999-51	*C.annuum*	Group 13	Type 5	N	N	N	N	N
C04398-A	*C.annuum*	Group 13	Type 5	N	N	N	N	N
Mulato	*C.annuum*	Group 14	Type 5	N	N	N	N	N
Numex Bailey Piquin	*C.annuum*	Group 15	Type 5	N	W	N	N	N
BukangA	*C.annuum* (CMS)	Group 12	Type 4	Y	Y	Y	Y	Y
Chungyang A	*C.annuum* (CMS)	Group 12	Type 4	Y	Y	Y	Y	Y
KA-2	*C.annuum* (CMS)	Group 12	Type 4	Y	Y	Y	Y	Y
Manitta	*C.annuum* (CMS)	Group 12	Type 4	Y	Y	Y	Y	Y
P 82005	*C. frutescens*	-	-	N	N	N	N	N
NUM-6 L.G.G.-J.L.	*C. frutescens*	-	-	N	N	N	W	N
C00683	*C. frutescens*	-	-	N	N	N	W	N
PI 555644	*C. frutescens*	-	-	N	N	N	N	N
GREEN LEAF VARIETY	*C. frutescens*	-	-	N	N	N	N	N
C 04750-B	*C. frutescens*	-	-	N	N	N	N	N
VTP 220	*C. frutescens*	-	-	N	W	N	W	N
C 04612	*C. frutescens*	-	-	N	N	N	W	N
KC01293	*C. frutescens*	-	-	N	N	N	W	N
Lombok	*C. frutescens*	-	-	N	N	N	N	N
AC 08-010	*C. frutescens*	-	-	N	N	N	N	N
C 04851	*C. frutescens*	-	-	N	N	N	N	N
KC01303	*C. frutescens*	-	-	N	N	N	N	N
KC01288	*C. frutescens*	-	-	N	N	N	N	N
Toko Subur	*C. frutescens*	-	-	Y	Y	Y	N	N
EKIRIKE	*C. frutescens*	-	-	N	N	N	W	N
chi 10/1011-2	*C. chinense*	-	-	N	N	N	N	N
AC 08-045	*C. chinense*	-	-	N	N	N	N	N
Avella	*C. chinense*	-	-	N	Y	N	N	N
C00643	*C. chinense*	-	-	N	N	N	W	N
C04417	*C. chinense*	-	-	N	N	N	N	N
chi 16/1028-1	*C. chinense*	-	-	N	N	N	N	N
chi 12/1021-2	*C. chinense*	-	-	N	Y	N	N	N
chi 12/1018	*C. chinense*	-	-	N	N	N	N	N
Hot Jalapeno	*C. chinense*	-	-	N	N	N	N	N
SAFI	*C. chinense*	-	-	N	Y	N	N	N
Locoto	*C. chinense*	-	-	N	N	N	N	N
chi 38/1051-2	*C. chinense*	-	-	N	N	N	N	N
chi 15/1024	*C. chinense*	-	-	N	N	N	W	N
chi 22b/1037-2	*C. chinense*	-	-	N	N	N	N	N
C01436	*C. chinense*	-	-	N	N	N	N	N
chi 22a/1036	*C. chinense*	-	-	N	N	N	W	N
C04220	*C. baccatum*	-	-	N	N	N	W	N
C 04065	*C. baccatum*	-	-	N	N	N	N	N
C01526	*C. baccatum*	-	-	N	N	N	N	N

C 04084	*C. baccatum*	-	-	N	N	N	W	N
Pimenta	*C. baccatum*	-	-	N	N	N	N	N
C 04069	*C. baccatum*	-	-	N	N	N	N	N
pen-1/19864/1	*C. baccatum*	-	-	N	N	N	N	N
C 04329	*C. baccatum*	-	-	N	N	N	N	N
CC3751	*C. baccatum*	-	-	N	N	N	W	N
C 02433	*C. baccatum*	-	-	N	N	N	W	N
C04221	*C. baccatum*	-	-	N	N	N	W	N
C04659-A	*C. baccatum*	-	-	N	N	N	N	N
ROSENPORRIKA E86	*C. baccatum*	-	-	N	N	N	W	N
AC 08-059	*C. baccatum*	-	-	N	N	N	N	N
C01543	*C. baccatum*	-	-	N	N	N	W	N
pen-40/19896/1	*C. baccatum*	-	-	N	N	N	W	N
